# Comparative efficacy of delafloxacin for complicated and acute bacterial skin and skin structure infections: results from a network meta-analysis

**DOI:** 10.1186/s12879-021-06736-x

**Published:** 2021-10-05

**Authors:** Ioanna Vlachaki, Matteo Vacchelli, Daniela Zinzi, Edel Falla, Yilin Jiang, Theo Mantopoulos, Dilip Nathwani

**Affiliations:** 1 Menarini Ricerche Spa, Florence, Italy; 2 Menarini Industrie Farmaceutiche Riunite, Via Sette Santi 1/3, 50131 Florence, Italy; 3 EMEA Real World Methods and Evidence Generation, IQVIA Ltd, London, UK; 4 EMEA Real World Methods and Evidence Generation, IQVIA Ltd, Amsterdam, Netherlands; 5 EMEA Real World Methods and Evidence Generation, IQVIA Ltd, Athens, Greece; 6 Emeritus Honorary Professor of Infection, Medical School, University of Dundee, Dundee, DD1 9SY UK

**Keywords:** Acute bacterial skin and skin structure infections, Methicillin-resistant *Staphylococcus aureus*, Delafloxacin, Network meta-analysis

## Abstract

**Background:**

Delafloxacin is a novel fluoroquinolone with broad antibacterial activity against pathogens causing acute bacterial skin and skin structure infections (ABSSSI). This network meta-analysis (NMA) was conducted to evaluate the relative efficacy of delafloxacin versus other comparators used for managing patients with ABSSSI.

**Methods:**

A systematic literature review was conducted to identify randomised controlled trials (RCTs) evaluating adults (≥ 18 years) with ABSSSI, complicated SSSI (cSSSI), complicated skin and soft tissue infections (cSSTI) or severe cellulitis with pathogen of gram-positive, gram-negative, or mixed aetiology. OVID MEDLINE^®^, Embase, Epub Ahead of Print, Cochrane Central Register of Controlled Trials and Cochrane Database of Systematic Reviews were searched from inception through 12 April 2019. A feasibility assessment was conducted, followed by an NMA, which was run in a Bayesian framework. The interventions included in the NMA encompassed monotherapy or combination therapies of amoxicillin/clavulanate, ampicillin/sulbactam, ceftaroline, ceftobiprole, dalbavancin, daptomycin, delafloxacin, fusidic acid, iclaprim, linezolid, omadacycline, oxacillin + dicloxacillin, standard therapy, tedizolid, telavancin, tigecycline, vancomycin, vancomycin + aztreonam and vancomycin + linezolid.

**Results:**

A feasibility assessment was performed and evidence networks were established for composite clinical response (n = 34 studies), early clinical response (n = 16 studies) and microbiological response (n = 14 studies) in the overall study population, composite clinical response (n = 4 studies) in obese subpopulation and for composite clinical response (n = 18 studies) and microbiological response (n = 14 studies) in patients with methicillin-resistant *Staphylococcus aureus* (MRSA) infection. Delafloxacin performed significantly better than fusidic acid, iclaprim, vancomycin, and ceftobiprole for composite clinical response. Delafloxacin was comparable to dalbavancin, daptomycin, fusidic acid, iclaprim, linezolid, omadacycline, tedizolid, vancomycin, vancomycin + aztreonam and vancomycin + linezolid in the analysis of early clinical response, whereas for microbiological response, delafloxacin was comparable to all interventions. In the obese subpopulation, the results favoured delafloxacin in comparison to vancomycin, whilst the results were comparable with other interventions among the MRSA subpopulation.

**Conclusions:**

Delafloxacin is a promising new antibiotic for ABSSSI demonstrating greater improvement (composite clinical response) compared to ceftobiprole, fusidic acid, iclaprim, telavancin and vancomycin and comparable effectiveness versus standard of care for all outcomes considered in the study.

**Supplementary Information:**

The online version contains supplementary material available at 10.1186/s12879-021-06736-x.

## Introduction

Complicated skin and soft tissue infections (cSSTI), or complicated skin and skin structure infections (cSSSI), represent a severe form of skin infections involving the skin and deeper soft tissues [1]. cSSSI have been associated with increasing morbidity, mortality and healthcare costs in recent decades [1, 2]. The overall incidence of SSTI in the United States (US) increased 40% from 2.4 million in 2000 to 3.3 million in 2012 [3]. In 2013, the US Food and Drug Administration (FDA) distinctly defined a subset of severe skin infections as acute bacterial skin and skin structure infections (ABSSSI) to facilitate the clinical development of drugs and evaluate efficacy of novel antibiotics through quantifiable variables such as lesion size and systemic signs of infections [4, 5]. According to the US FDA, ABSSSI include cellulitis, erysipelas, wound infections or major cutaneous abscesses with a minimum lesion surface area of 75 cm^2^, measured by erythema, oedema or induration [5, 6]. ABSSSI are caused most commonly by gram-positive bacteria such as *Streptococcus pyogenes* and *Staphylococcus aureus*, including methicillin-resistant *S. aureus* (MRSA) strains [5, 7, 8]. In 2018, epidemiological data from 30 participating countries in the European Antimicrobial Resistance Surveillance (EARS) network and European Centre for Disease prevention and Control (ECDC) estimated that MRSA accounted for 16.4% of all *S. aureus* isolates, with significant differences in national MRSA percentages ranging from 0% in Iceland to 43% in Romania [9]. The Centre for Disease Control and Prevention has recognised MRSA as a serious health threat to humans with an estimated 323,700 hospitalisations and 10,600 deaths across the US in 2017 [10]. Despite a decline in MRSA- related hospitalisations since 2005, MRSA accounts for significant morbidity and mortality in the US [11, 12].

Treatment approaches for ABSSSI according to the Infectious Disease Society of America (IDSA) practice guidelines include surgical drainage or debridement when appropriate, culture and susceptibility testing and appropriate empiric antibiotic therapy [6]. The increasing prevalence of MRSA in the past decade has altered the therapeutic approach to ABSSSI [6, 13]. The IDSA guidelines recommend vancomycin as the first-line agent for MRSA ABSSSI [6]. However, higher vancomycin doses often used to treat MRSA infections may lead to serious complications such as nephrotoxicity, ototoxicity and hypersensitivity reactions, which can be associated with prolonged hospitalisation and increased healthcare costs [14–17]. Other recommended antibiotics for MRSA include linezolid, daptomycin, ceftaroline and telavancin [6]. However, these antibiotics are ineffective against gram-negative bacteria, except ceftaroline which is effective against some gram-negative pathogens, excluding *Pseudomonas aeruginosa* and multi-drug resistant gram-negative bacteria [18]. Nafcillin, cefazolin and clindamycin may also be considered for ABSSSI caused by methicillin-sensitive *S. aureus* (MSSA) [6, 19]. The antimicrobial activity of recently approved antibiotics for the treatment of ABSSSI, namely, dalbavancin [20], oritavancin [21] and tedizolid [22] are also limited to strains of gram-positive pathogens, including MRSA. The global emergence of MRSA strains and other resistant pathogens in recent years has imposed a considerable challenge to its management, compelling a drive to develop safe and effective antibiotics for the treatment of ABSSSI, including MRSA-related infections [9, 23].

Obese patients with ABSSSIs are at a higher risk of treatment failure and have demonstrated slow recovery [24, 25] According to the World Health Organisation, globally 39% adults (≥ 18 years) were overweight, whilst about 13% of the world’s adult population was obese in 2016, making it a clinically important subpopulation of interest [26].

Delafloxacin, a novel fluoroquinolone with activity against a diverse range of gram-positive (*S. aureus,*[including MRSA] and *Streptococcus pyogenes*) and gram-negative bacteria (*Pseudomonas aeruginosa, Escherichia coli* and *Klebsiella pneumoniae*), was approved by the US FDA for ABSSSI in 2017 and by the European Medicines Agency in 2019 [27–29]. Delafloxacin is available in oral and intravenous (IV) forms, at a recommended dose of 300 mg IV or 450 mg orally once every 12 h [28]. The clinical efficacy of delafloxacin was explored in a large developmental programme that included two phase III clinical trials, demonstrating that delafloxacin was non-inferior to the combination of vancomycin plus aztreonam for the treatment of ABSSSI [30, 31]. Whilst the efficacy of delafloxacin in ABSSSI was assessed in comparison to vancomycin plus aztreonam, there are no randomised controlled trials (RCTs) available to assess the comparative effectiveness for other comparators used for the management of ABSSSI.

Two previous systematic literature reviews (SLRs) and meta-analyses investigating the safety and efficacy of delafloxacin for the treatment of ABSSSI in adult patients demonstrated similar clinical cure rates of delafloxacin versus comparators in the treatment of ABSSSI and MRSA ABSSSI’s [32, 33]. However, these studies included publications with ABSSSI populations only and as such, evaluated overall clinical cure or microbiological response for limited number of interventions (i.e. ceftobiprole, linezolid, tigecycline, and vancomycin/aztreonam) among the current standard of care for the treatment of ABSSSI. This network meta-analysis (NMA) aims to add to the existing knowledge on the performance of delafloxacin in terms of composite clinical response, microbiological response and early response versus the current standard of care, including obese patients (body mass index [BMI] ≥ 30 kg/m^2^) and patients with MRSA.

## Methods

An SLR was conducted in accordance with the Cochrane Handbook for Systematic Reviews of Interventions Version 5.1.0 to identify the clinical evidence from RCTs for delafloxacin versus standard of care in adult patients with ABSSSI, cSSSI, cSSTI or severe cellulitis [34].

### Data sources and search strategy

OVID MEDLINE^®^, Embase, Epub Ahead of Print (In-process & other non-indexed citations), and Cochrane Central Register of Controlled Trials and Cochrane Database of Systematic Reviews were searched for relevant RCTs with a prespecified search strategy. Additional searches were performed in the conference proceedings of European Society of Clinical Microbiology and Infectious Diseases, Infectious Diseases Week and World Antimicrobial Resistance Congress Europe from 2017 to 2019. The searches executed in the SLR were based on pre-defined patient population, intervention, comparators, outcome measures and study design criteria (Additional file 1: Appendix A). RCTs involving adult patients with ABSSSI, cSSSI, cSSTI or severe cellulitis, were selected for inclusion. The databases were systematically searched from inception through 12 April 2019.

### Study selection and identification

All identified records were exported to Microsoft^®^ Excel after removing duplicates in a reference management software. Abstract screening was conducted based on pre-defined eligibility criteria. Publications with uncertainty were reviewed by an independent second reviewer, and any disagreement was resolved either through “reconciliation” (discussion between the two reviewers) or through “arbitration” with a third independent reviewer where “majority view” determined the inclusion/exclusion. All records included at the end of this stage were retained for full text review, followed by data extraction and quality appraisal. A descriptive quality assessment of the included RCTs was performed using the Cochrane checklist as per the National Institute for Health and Care Excellence (NICE) technical support document (TSD) 2 for NMA of RCTs of RCTs (Additional file 1: Appendix B) [35, 36].

### Feasibility assessment

The feasibility of conducting an NMA was examined by first assessing if a connected network of evidence could be established for each outcome of interest, based on the clinical evidence identified from the SLR. The studies included in the connected networks were further assessed for the presence and extent of between-study heterogeneity. To assess the comparability of study populations, a comparison of patients’ baseline characteristics (i.e. age, gender, BMI, race, MRSA/MSSA population, treatment duration, polymicrobial infections, and comorbidities) was conducted. Study design characteristics for all included RCTs (e.g. cross-over or open label) were assessed to identify potential sources of bias that could impact the outcomes of interest (Additional file 1: Appendix C).

### Evidence synthesis assumptions

#### Assumptions on study outcomes

Due to the heterogeneity of outcome definitions, studies with similar outcome definitions at similar timepoints were grouped together, and any assumptions were validated by two clinical experts (Additional file 1: Appendix D).

The RCTs reporting clinical cure or success outcomes at the end of therapy, test of cure (TOC), post-therapy evaluation or follow-up were considered for the outcome ‘composite clinical response’ in the analysis. Wherever a study reported both clinical cure and clinical success, clinical success was considered as the efficacy outcome for analysis. ‘Early response’ was determined as either ≥ 20% lesion size reduction or early clinical response at 48–72 h. When a study reported both ≥ 20% lesion size reduction and early clinical response, ≥ 20% lesion size reduction was considered as the efficacy outcome for analysis. Documented eradication or presumed eradication of baseline pathogens was considered as ‘microbiological response’.

#### Assumptions on the study design and patient population

The potential bias due to any heterogeneity in study design and patient characteristics were also investigated and validated by a clinical expert. Populations and study design were deemed to be comparable and unlikely to have an impact on the NMA results. Variation in antibiotic dosing across trials was observed for delafloxacin, telavancin, iclaprim, vancomycin, omadacycline and dalbavancin, however as per the clinical expert opinion the variation in dosing schedules between studies was unlikely to influence the NMA results (Additional file 1: Appendix D). Doses were therefore pooled in the analysis after consultation with two clinical experts. For each study, outcome data from the ITT population were used in the analysis, whereas in the absence of the ITT population for a particular study, data from the population closest to the ITT was used instead (e.g., modified ITT). The analysis population from each RCT is presented in supplementary material Additional file 1: Appendix D.

### Network meta-analysis

Bayesian NMA models were used to synthesise the results of included studies as per the NICE guidance [36]. The analyses were based on a burn-in of 80,000 iterations and a further sample of 20,000 iterations until convergence was achieved. The Monte Carlo error was captured, which reflects both the number of simulations and the degree of autocorrelation. This should be no more than 5% of the posterior standard deviation of the parameters of interest. Finally, visual inspection of trace/density plots was carried out. As suggested by the NICE Decision Support Unit TSD 2 a normal distribution with zero mean and variance equal to 10^4^ was used for treatment effects and a uniform distribution with range zero to 5 for the between-trial standard deviation [36]. Vague (flat/uninformative) priors were used for all calculations. The analyses were conducted using JAGS (version 4.3.0) with RStudio (version 3.5.1) as the front end. The analyses consisted of binary outcomes. A binomial model with a logit link function was employed for all outcomes based on NICE guidance [36]. Both fixed effects and random effects models were run to test the model fit and assess heterogeneity. Given that the fixed effect models provided better fit than the random effect models in all analyses and the limited information to estimate between-study variance in the random effect models in some analyses, only results for the fixed effects models are discussed in the main body of the paper. Model fit statistics and results from the random effect models are presented in Additional file 1: Appendix E and Appendix F, respectively. Inconsistency, that is, the lack of agreement between direct and indirect evidence in an NMA, was assessed for each loop in the networks. Inconsistency was assessed by the inconsistency model, as described in the NICE Decision Support Unit TSD 4 [37].

For each outcome, an odds ratio (OR) was used to reflect the relative treatment effects between interventions. Forest plots were presented using the posterior median of OR for each pairwise treatment comparison. The 2.5th and 97.5th percentiles to capture the 95% credible interval (CrI) of corresponding ORs were also provided along with the posterior median. For all outcomes a median OR > 1 indicates favourable results for delafloxacin.

## Results

**Fig. 1:**
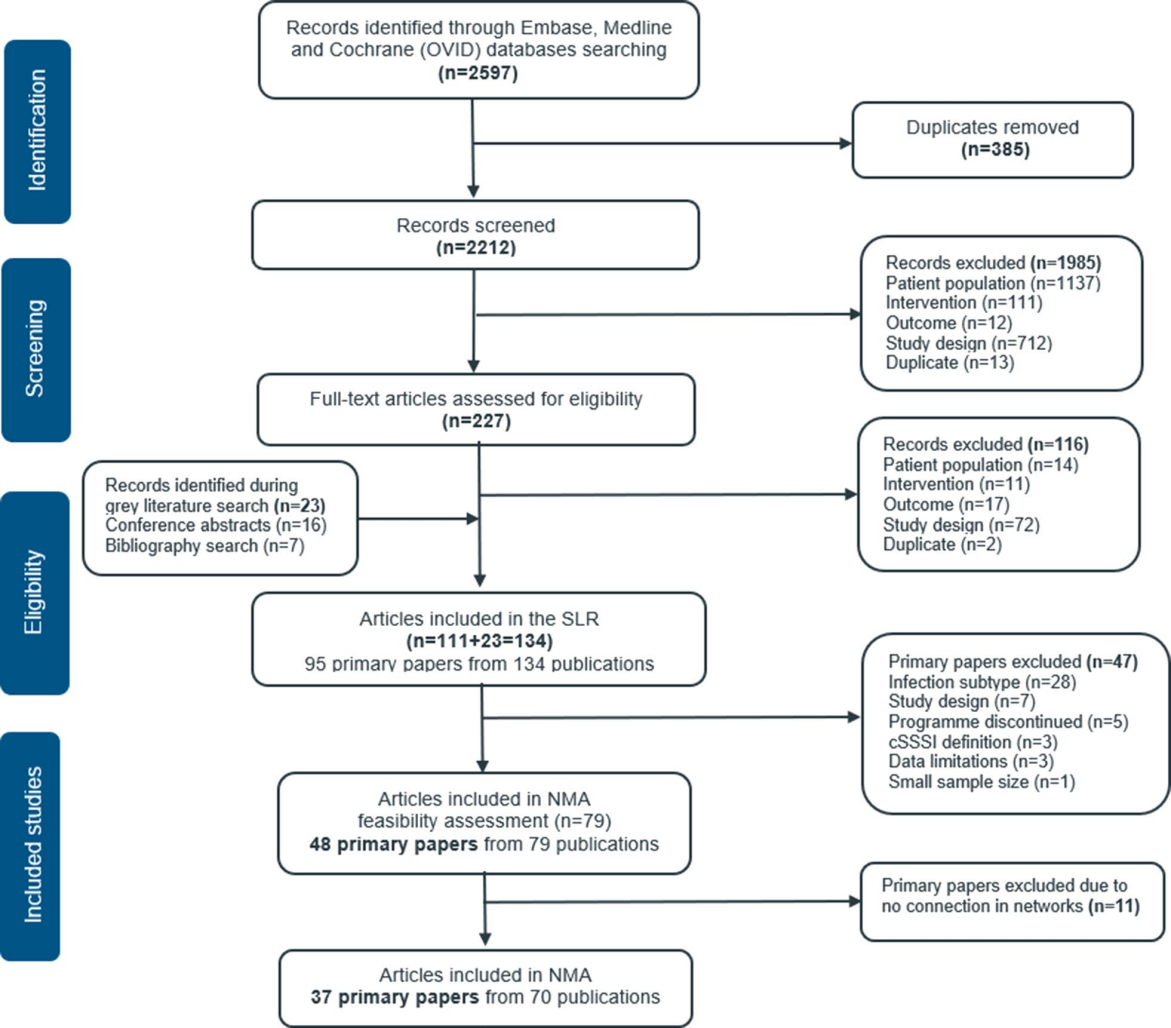
PRISMA flow chart. *NMA* network metanalysis, *SLR* systematic literature review

The SLR search identified 2212 studies after removing duplicates. Following the abstract screening, 1985 studies were excluded as they did not meet the eligibility criteria and 227 publications were assessed for full text review. Out of these, 48 primary studies from 79 publications were considered for the NMA feasibility assessment. Nine primary studies were excluded as they did not form a connection in the networks. Two oritavancin phase III RCTs were excluded from the NMA, as the study population in these two studies included a lower proportion of elderly and obese patients than the pivotal RCT on delafloxacin [38, 39]. Overall, 37 primary RCTs were included in the NMA following full text screening and feasibility assessment (Fig. 1). The results of the quality assessment for included RCTs suggested an overall low or moderate risk of bias. A high risk of bias was observed in terms of blinding for seven open label RCTs [40–46]. Evidence networks were established for the overall study population (outcomes: composite clinical response, early clinical response, and microbiological response), patients with obesity (outcome: composite clinical response) and patients with MRSA (outcomes: composite clinical response and microbiological response). The evidence network for patients with obesity (outcomes: early clinical response and microbiological response) and patients with MRSA (outcome: early clinical response) could not be established due to the limited number of studies reporting outcomes for these subgroups.

An assessment of the degree of between-study heterogeneity was conducted and was validated with two clinical experts by examining differences across studies eligible for inclusion in the NMA. Potential effect modifiers were generally similar across the included studies, and where variability across studies was noted (e.g., proportion of MSSA patients, proportion of MRSA patients, mean treatment duration, proportion of patients with comorbidities), they were further discussed and validated with clinical experts. The potential sources of heterogeneity are presented in Additional file 1: Appendix C. The outcome of the feasibility was that the studies were generally comparable with a low risk of bias from treatment effect modifiers. Based on feedback from the two clinical experts, and given that no considerable evidence of heterogeneity was found (as evidenced from the better fit of the fixed effect models over the random effect models), two sensitivity analyses were also conducted to assess the impact of the populations considered and three ‘outlier studies’ identified. The first sensitivity analysis included only studies which reported outcomes in the ITT (or mITT) population, and the second removed three studies (Wilcox et al. 2009 [42], Sharpe et al. 2005 [46] and Pushker et al. [47]) from the evidence networks, which were deemed to potentially introduce bias due to their small sample sizes and differences in baseline patient characteristics compared to the other included studies.

Inconsistency was tested in the evidence networks for “all patients: composite clinical response” and “all patients: microbiological response”, as these two networks had closed loops (between delafloxacin, vancomycin, vancomycin + aztreonam, ceftaroline and tigecycline in the first network and between delafloxacin, vancomycin, vancomycin + aztreonam, ceftaroline in the second). No evidence of inconsistency in either network was found. Results from the inconsistency assessment are presented in Additional file 1: Appendix G.

Across the 37 RCTs in the evidence networks, efficacy outcomes were included from 18 interventions, of which vancomycin and linezolid were the most common interventions (Additional file 1: Appendix D). Two studies considered the use of either an anti-staphylococcal penicillin (including activity against MRSA) or vancomycin as ‘Standard therapy’ (ST) [48, 49].

### All patients

#### Composite clinical response

**Fig. 2:**
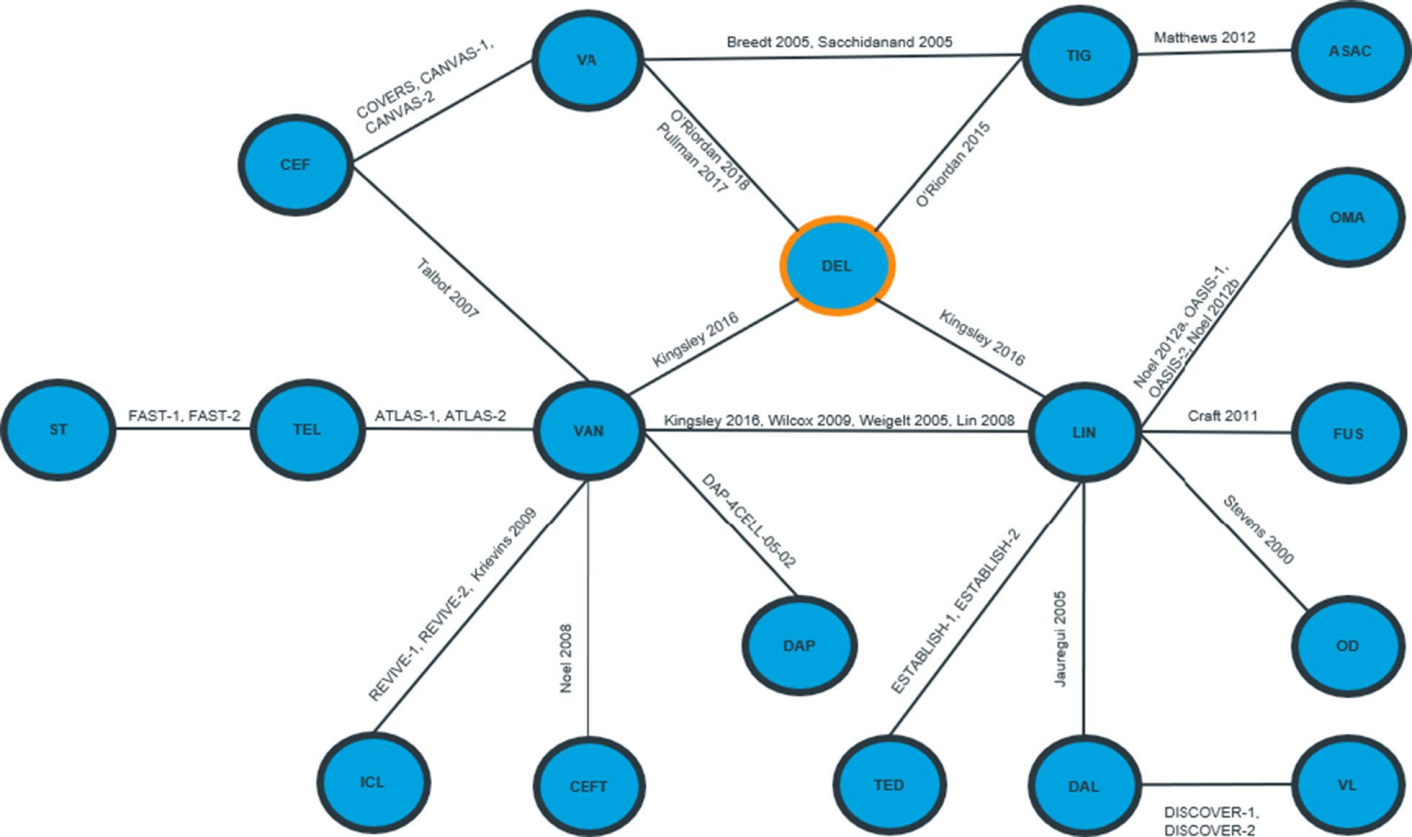
Network of evidence for all patients: composite clinical response. *ASAC* ampicillin/sulbactam or amoxicillin/clavulanate, *CEF* ceftaroline, *CEFT* ceftobiprole, *DAL* dalbavancin, *DAP* daptomycin, *DEL* delafloxacin, *FUS* fusidic acid, *ICL* iclaprim, *LIN* linezolid, *OD* oxacillin + dicloxacillin, *OMA* omadacycline, *ST* standard therapy, *TED* tedizolid, *TEL* telavancin, *TIG* tigecycline, *VA* vancomycin + aztreonam, *VAN* vancomycin, *VL* vancomycin + linezolid

**Fig. 3:**
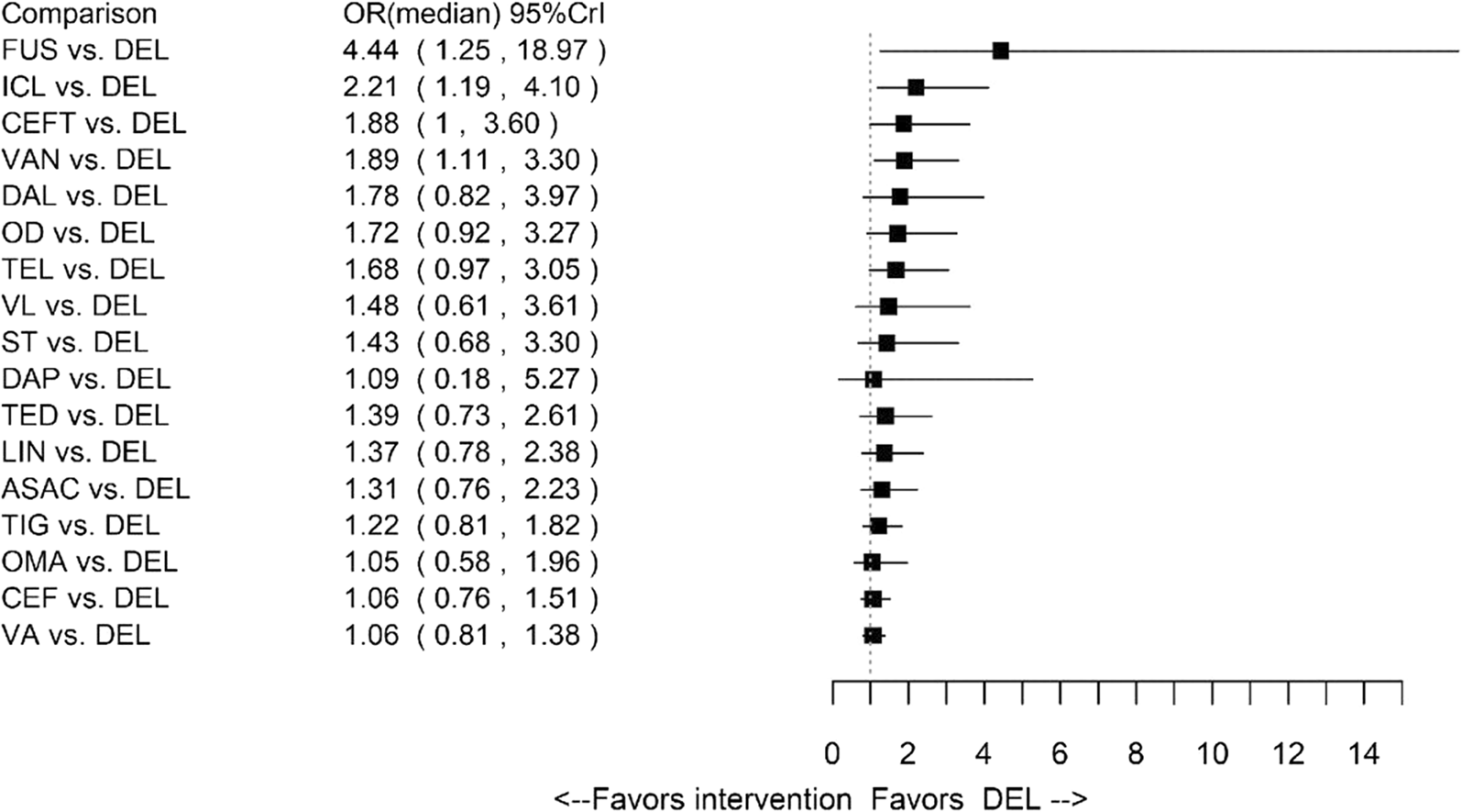
Forest plot for all patients: composite clinical response. *ASAC* ampicillin/sulbactam or amoxicillin/clavulanate, *CEF* ceftaroline, *CEFT* ceftobiprole, *DAL* dalbavancin, *DAP* daptomycin, *DEL* delafloxacin, *FUS* fusidic acid, *ICL* iclaprim, *LIN* linezolid, *OD* oxacillin + dicloxacillin, *OMA* omadacycline, *ST* standard therapy, *TED* tedizolid, *TEL* telavancin, *TIG* tigecycline, *VA* vancomycin + aztreonam, *VAN* vancomycin, *VL* vancomycin + linezolid

The network of evidence for composite clinical response consisted of 34 studies, reporting estimates for 18 interventions (Fig. 2). The results were in favour of delafloxacin in comparison to ceftobiprole, fusidic acid, iclaprim and vancomycin. Results for amoxicillin/clavulanate, ampicillin/sulbactam, ceftaroline fosamil, dalbavancin, daptomycin, delafloxacin, linezolid, omadacycline, oxacillin + dicloxacillin, ST (i.e. anti-staphylococcal penicillin or vancomycin), tedizolid, telavancin, tigecycline, vancomycin + aztreonam and vancomycin + linezolid were comparable to delafloxacin. The forest plot of median ORs and associated 95% CrIs for delafloxacin versus all comparators is presented in Fig. 3.

### Early clinical response

**Fig. 4:**
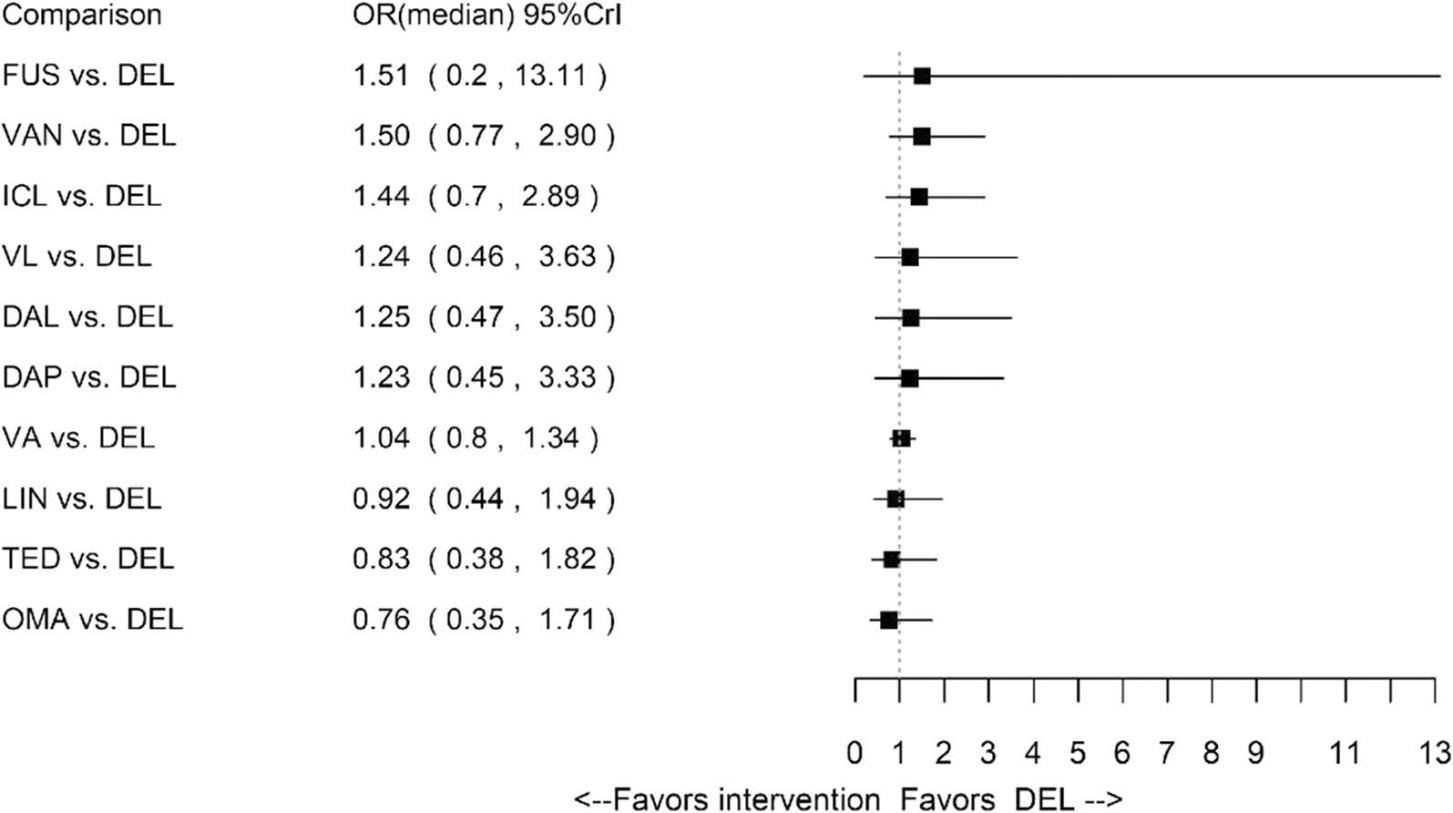
Forest plot for all patients: early clinical response. *DAL* dalbavancin, *DAP* daptomycin, *DEL* delafloxacin, *FUS* fusidic acid, *ICL* iclaprim, *LIN* linezolid, *OMA* omadacycline, *TED* tedizolid, *VA* vancomycin + aztreonam, *VAN* vancomycin, *VL* vancomycin + linezolid

In terms of early clinical response, delafloxacin was comparable to dalbavancin, daptomycin, fusidic acid, iclaprim, linezolid, omadacycline, tedizolid, vancomycin, vancomycin + aztreonam and vancomycin + linezolid. The network of evidence and the forest plot of median ORs and associated 95% CrIs for early clinical response are presented in Additional file 1: Appendix H and Fig. 4, respectively.

### Microbiological response

**Fig. 5:**
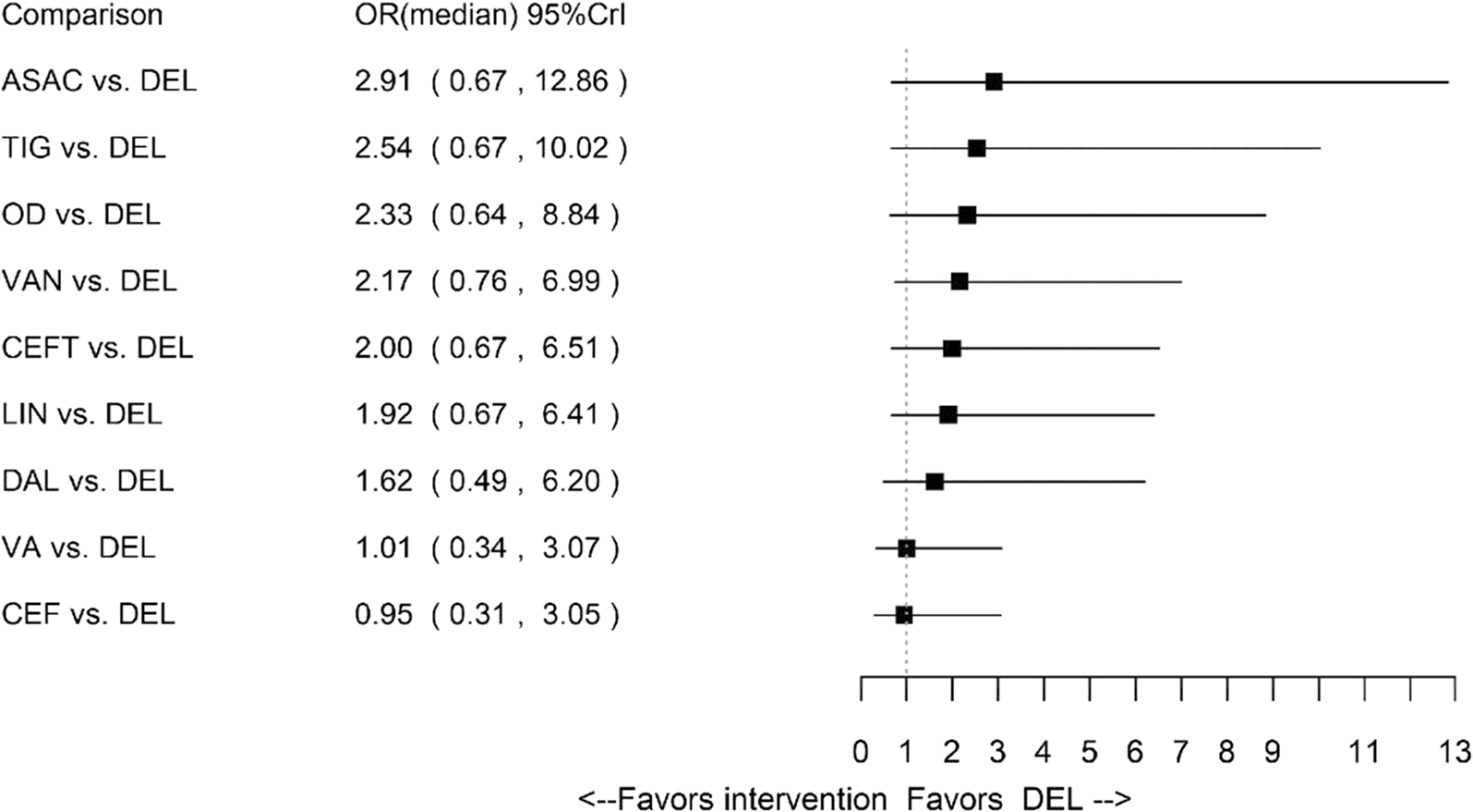
Forest plot for all patients: microbiological response. *ASAC* ampicillin/sulbactam or amoxicillin/clavulanate, *CEF* ceftaroline fosamil, *CEFT* ceftobiprole, *DAL* dalbavancin, *DEL* delafloxacin, *LIN* linezolid, *OD* oxacillin + dicloxacillin, *TIG* tigecycline, *VA* vancomycin + aztreonam, *VAN* vancomycin

For microbiological response, delafloxacin was comparable to all interventions i.e. ampicillin/sulbactam, amoxicillin/clavulanate, ceftaroline, ceftobiprole, dalbavancin, linezolid, oxacillin + dicloxacillin, tigecycline, vancomycin + aztreonam, and vancomycin. The median ORs and associated 95% CrIs for microbiological response are presented in Fig. 5 and the network of evidence is presented in Additional file 1: Appendix I.

### Obese patients (BMI ≥ 30 kg/m^2^)

#### Composite clinical response

**Fig. 6:**
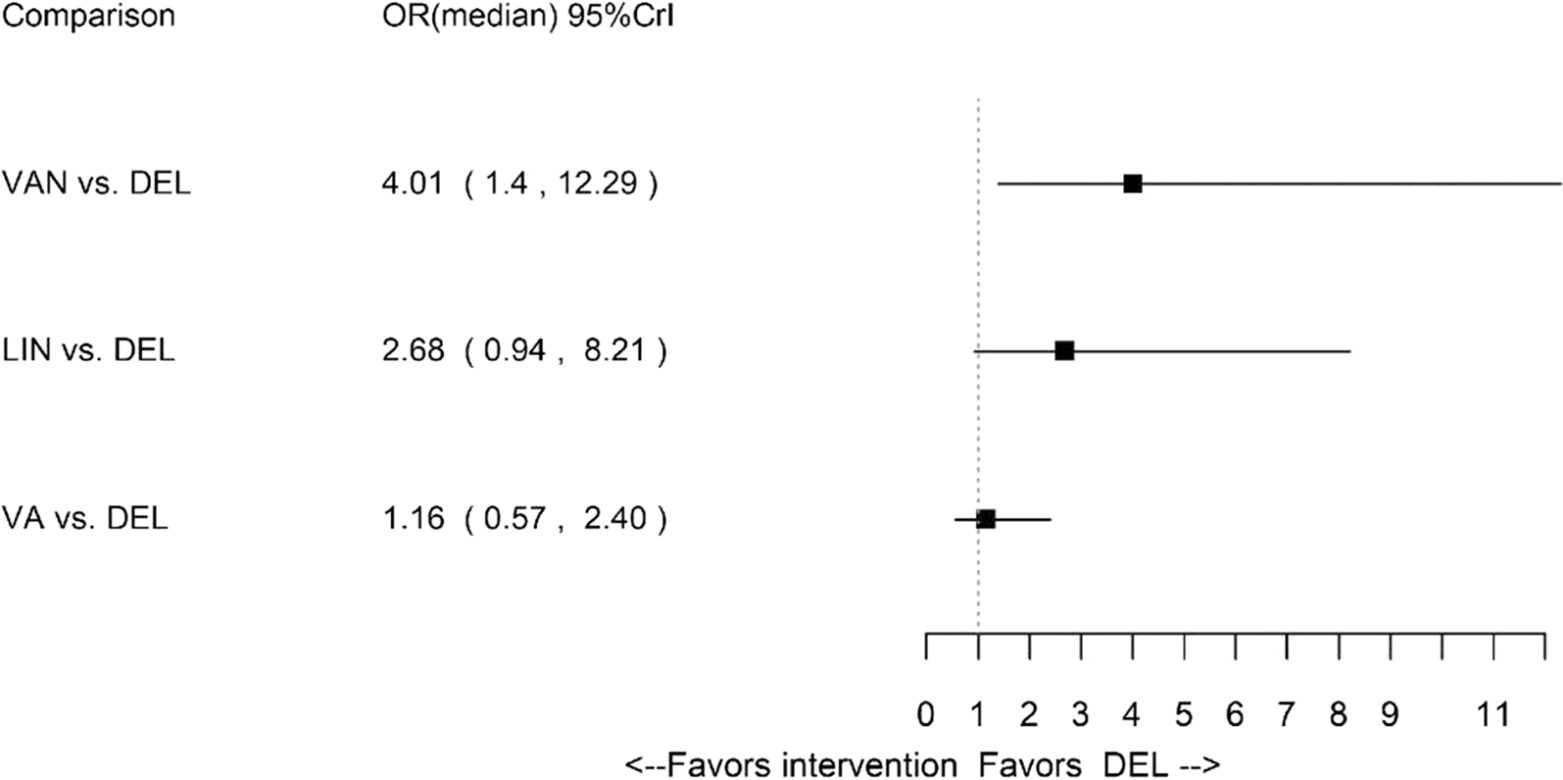
Forest plot for obese subpopulation: composite clinical response. *DEL* delafloxacin, *Lin* linezolid, VA vancomycin + aztreonam, *VAN* vancomycin

Results for composite clinical response in obese patients favoured delafloxacin in comparison to vancomycin. However, the results for delafloxacin, linezolid and vancomycin + aztreonam were comparable. The forest plot of median ORs and associated 95% CrIs in obese patients is presented in Fig. 6, and the network of evidence is presented in Additional file 1: Appendix J.

#### Additional subgroup: patients with MRSA infection

The network of evidence for patients with MRSA infection consisted of 18 studies, reporting estimates for ten interventions. For composite clinical response, delafloxacin was found to be comparable to all interventions in the network, namely, ampicillin/sulbactam, amoxicillin/clavulanate, ceftobiprole, ceftaroline, linezolid, omadacycline, tigecycline, vancomycin + aztreonam and vancomycin (Additional file 1: Appendix K). Similarly, for microbiological response, delafloxacin was found to be comparable to ceftaroline, dalbavancin, linezolid, tedizolid, tigecycline, vancomycin + aztreonam and vancomycin (Additional file 1: Appendix L).

### Sensitivity analyses

#### Sensitivity analyses for ITT/mITT population

Evidence networks were established for the overall population for composite clinical response and early clinical response. Delafloxacin was superior in terms of composite clinical response in comparisons with fusidic acid, iclaprim, vancomycin, and ceftobiprole, whilst with other active comparators results were comparable with delafloxacin. In early clinical response the results were comparable for delafloxacin versus fusidic acid, vancomycin, iclaprim, omadacycline, linezolid, tedizolid, and vancomycin + aztreonam.

#### Sensitivity analyses after removing outlier studies

Evidence networks were established for the overall population for composite clinical response and microbiological response. For composite clinical response, in comparisons of delafloxacin with fusidic acid, iclaprim, vancomycin, ceftobiprole, and telavancin, the results favoured delafloxacin. For the comparisons of delafloxacin with the remaining treatments, the results were comparable between delafloxacin and the comparator treatments.

For microbiological response, the results were comparable for delafloxacin versus ampicillin + sulbactam or amoxycillin + clavulanate, ceftobiprole, dalbavancin, linezolid, oxacillin + dicloxacillin, tigecycline, vancomycin, ceftaroline and vancomycin + aztreonam.

## Discussion

Delafloxacin is a fluoroquinolone with broad spectrum activity against gram-positive pathogens, including MRSA and many gram-negative pathogens [30, 50]. This NMA included RCTs involving adult patients with ABSSSI, cSSSI, cSSTI or severe cellulitis and comprehensively evaluated the relative clinical efficacy of delafloxacin versus interventions used to treat patients with complicated and ABSSSI. In comparison to previously published NMAs, this NMA included a broader range of infections such as ABSSSI, cSSSIs, cSSTI or severe cellulitis, together with a wider array of interventions [32, 33].

The NMA found that, for composite clinical response in the overall patient population, the results were in favour of delafloxacin when compared to ceftobiprole, fusidic acid, iclaprim, and vancomycin. For early clinical response, the results were comparable for delafloxacin versus dalbavancin, daptomycin, fusidic acid, iclaprim, linezolid, omadacycline, tedizolid, vancomycin, vancomycin + aztreonam and vancomycin + linezolid. For microbiological response, delafloxacin showed comparable effectiveness with ampicillin/sulbactam, amoxicillin/clavulanate, ceftaroline, ceftobiprole, dalbavancin, linezolid, oxacillin + dicloxacillin, tigecycline, vancomycin + aztreonam, and vancomycin. The relative clinical efficacy of delafloxacin versus other treatments in obese patients demonstrated consistency with the results of composite clinical response in the overall population, as delafloxacin showed greater improvement in comparison to vancomycin, and comparable results with linezolid and vancomycin + aztreonam. Furthermore, delafloxacin showed comparable results with other included interventions in terms of composite clinical and microbiological response for MRSA patients. The sensitivity analysis including only trials reporting outcomes for the ITT/mITT populations and removing the three outlier studies identified by clinical experts were comparable to the base case analysis.

Across all analysed populations, particularly for the MRSA subpopulation, median OR estimates for several treatment comparisons were associated with considerable uncertainty, depicted by wide CrIs. This uncertainty can be attributed to the availability of data from a limited number of studies and heterogeneity in study populations. Therefore, robust conclusions cannot be derived for the MRSA subgroup analysis. It should be noted that as majority of the identified studies were not carried out exclusively in the MRSA population, these studies were not specifically designed or powered to detect differences in the subgroups. Furthermore, the proportion of patients with MRSA also varied across RCTs, which reflects the inherent heterogeneity between the included trials.

Our findings are consistent with a previous NMA involving four RCTs by Lan et al*.*, in which delafloxacin exhibited clinical cure rate similar to other comparator drugs (OR: 1.05; 95% CI: 0.87 to 1.27; I^2^ = 16%) in the treatment of ABSSSI [32]. Although, Lan et al*.* included an adult population with ABSSSI that was limited to delafloxacin RCTs, thereby comparing delafloxacin to just four comparators i.e. tigecycline, vancomycin, linezolid and vancomycin + aztreonam [32]. In another NMA which included ten RCTs, the indirect comparison of delafloxacin showed similar efficacy in terms of clinical cure with ceftaroline (OR: 0.82; 95% CrI: 0.39 to 1.8), ceftobiprole (OR: 0.79; 95% CrI: 0.32 to 1.9) and tigecycline (OR: 1.0: 95% CrI: 0.45 to 2.2) [33]. However, the analysis was restricted to the MRSA ABSSSI population and compared the efficacy of delafloxacin with just ceftaroline, ceftobiprole and tigecycline [33].

The present NMA encompassed 37 RCTs which included adult patients with a wide range of severe skin and skin structure infections. It evaluated the comparative effectiveness of delafloxacin versus 18 interventions in terms of composite clinical response, early clinical response and microbiological response. This NMA also evaluated the efficacy of delafloxacin with relevant comparators for obese patients, which to our knowledge has not been evaluated in previously published NMAs [32, 33]. In the present NMA, an assessment of the risk of bias was undertaken for each identified RCT and the variability across studies was validated by the clinical expert.

This study is subject to the limitations inherent to all NMAs in terms of heterogeneity of included studies [51]. The included RCTs were conducted in populations that were categorised by different definitions of infections (ABSSSI, cSSSI, cSSTI, severe cellulitis) and outcomes. This could lead to potential bias in the results. However, the assessment of inconsistency in the NMA did not suggest any discrepancy in the evidence. In addition, the studies included in the NMA were published over a time span extending two decades (2000–2019). A number of the included studies were conducted before the publication of the FDA guidance for the design of RCTs to evaluate drugs for ABSSSI [5], justifying the variability in the definition of infections. The RCTs assessing ceftaroline [52–54] and one of the RCTs assessing delafloxacin [55] considered patients with cSSSI whereas three RCTs for delafloxacin included patients with ABSSSI [30, 31, 56]. Moreover, different dosing regimens were used in RCTs assessing ceftaroline, dalbavancin, daptomycin, delafloxacin, linezolid, omadacycline, and telavancin. To facilitate the NMA, different doses of treatments were pooled for each study. However, the clinical expert validated that the treatment doses and infection subtypes across RCTs were comparable, therefore, these differences are unlikely to introduce bias in the results.

This study provides substantial indirect evidence for the comparative efficacy of delafloxacin versus a broad range of comparators for the management of ABSSSI. Further research is warranted for the comparative efficacy of delafloxacin involving RCTs powered to detect differences in populations with obesity and MRSA infections.

## Conclusion

The results of this NMA substantiate that delafloxacin is an effective new antibiotic for ABSSSI. Delafloxacin demonstrated improved composite clinical response versus ceftobiprole, fusidic acid, iclaprim and vancomycin in base case analysis, and with telavancin in the scenario analysis. For the remaining comparators in composite clinical response, in addition to all interventions included in the early response and microbiological response analysis, delafloxacin was equivalent. The results favoured delafloxacin in comparison to vancomycin for composite clinical response in obese patients. Finally, the NMA showed that delafloxacin has comparable efficacy to all interventions in the MRSA subgroup. Delafloxacin, with a broad spectrum activity against MRSA and gram-negative bacteria, is a promising addition to the standard of care for patients with ABSSSI.

## Supplementary Information

**Additional file 1.** Appendices.

## Data Availability

All data sets are provided in Additional file 1

## Abbreviations

ABSSSI: Acute bacterial skin and skin structure infections
BMI: Body mass index
Crl: Credible interval
cSSSI: Complicated skin and skin structure infections
cSSTI: Complicated skin and soft tissue infections
EARS: European Antimicrobial Resistance Surveillance
ECDC: European Centre for Disease prevention and Control
FDA: Food and Drug Administration
IDSA: Infectious Disease Society of America
IV: Intravenous
ITT: Intention-to-treat
MRSA: Methicillin-resistant *Staphylococcus aureus*
MSSA: Methicillin-sensitive *Staphylococcus aureus*
NICE: National Institute for Health and Care Excellence
NMA: Network meta-analysis
OR: Odds ratio
RCT: Randomised controlled trials
SLR: Systematic literature reviews
SSSI: Skin and skin structure infections
ST: Standard therapy
TOC: Test of cure
US: United States
