# Supplementary material for: Comparative efficacy of delafloxacin for complicated and acute bacterial skin and skin structure infections: results from a network meta-analysis
Source: BMC Infect Dis. 2021 Oct 5;21:1036. doi: 10.1186/s12879-021-06736-x (PMC8491383; doi:10.1186/s12879-021-06736-x)
Supplement: Supplementary file 1 — Additional file 1. Appendices. [file 12879_2021_6736_MOESM1_ESM.docx]

# **Additional File 1**

## **Appendix A: PICOS Table**

| **Domain** | **Inclusion criteria** | **Exclusion criteria** |
| --- | --- | --- |
| Population | - Adult patients (≥18 years) - Patients with ABSSSI, cSSSI, cSSTI or severe cellulitis - Infections of gram-positive/negative or mixed aetiology | - Patients aged <18 years - Healthy volunteers - Disease other than disease of our interest |
| Interventions | Monotherapy or combination therapy with any of the following:   - amoxicillin-clavulanic acid, aztreonam, ceftaroline fosamil, ceftriaxone, clindamycin, dalbavancin, daptomycin, delafloxacin (Quofenix®), ertapenem, etimicin, flucloxacillin, gepotidacin, linezolid, meropenem, oxacillin, piperacillin, tazobactam, tedizolid, teicoplanin, telavancin, tigecycline and vancomycin | - Intervention other than listed for inclusion - Non-pharmacological therapy |
| Comparators | - Any pharmacological therapy | - Non-pharmacological therapy |
| Outcomes | - Efficacy: - Clinical cure at follow-up - Clinical success at follow-up - Objective response based on ≥20% reduction in lesion erythema area at follow-up - Early response - Microbiological response at TOC - Relapse or recurrence - Readmission to hospital or re-hospitalisation - Hospital infection-related length of stay - Hospital length of stay - Safety: - ≥ 3/4 adverse events (nausea, diarrhoea etc.) OR - Serious adverse events   Data on following subgroups was extracted:   - MRSA - BMI ≥30 kg/m^2^ | - Outcomes other than listed for inclusion |
| Study design | - RCTs (no restriction on blinding status) | - Non-RCTs - Single arm trials - Observational studies - Non-systematic reviews - News, editorials, letters, comments - Case reports, case series |
| Restrictions | - Articles published in English language | - Articles published in non-English language |

^ABSSSI, acute bacterial skin and skin structure infections; cSSSI, complicated skin and skin structure infection; LOS, length of stay; RCTs, randomised controlled trials; SSSI, skin and skin structure infections; SSTI, skin and soft tissue infections; TOC, Test of Cure^

## **Appendix B: Quality assessment of included studies**


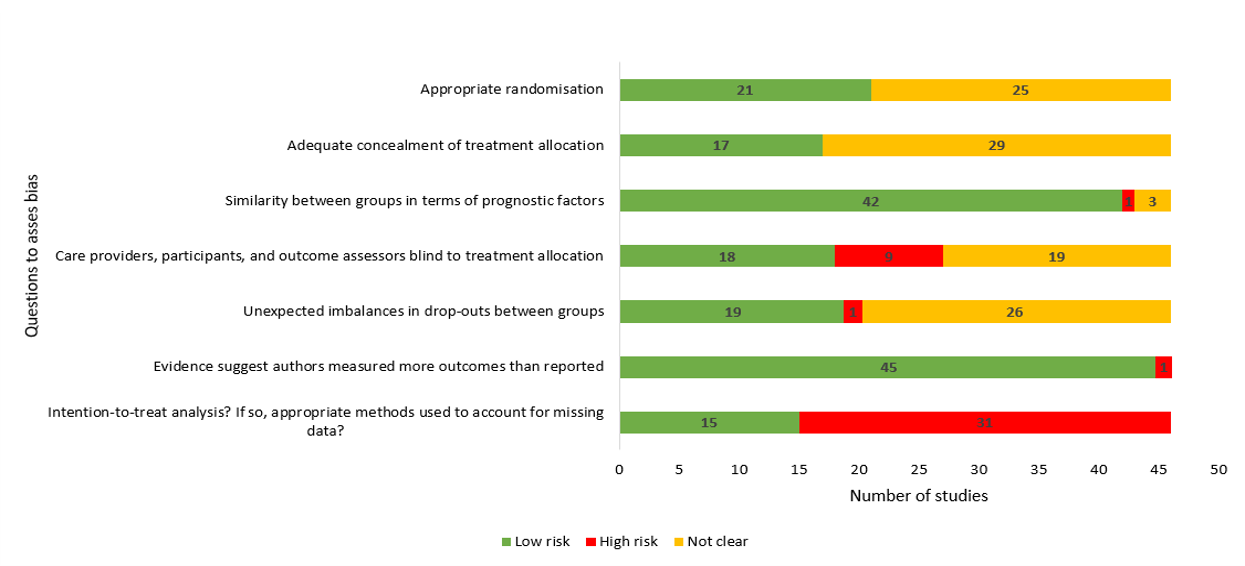


## **Appendix C: Sources of heterogeneity across trials identified from the SLR**

Appendix C1: Mean age in years


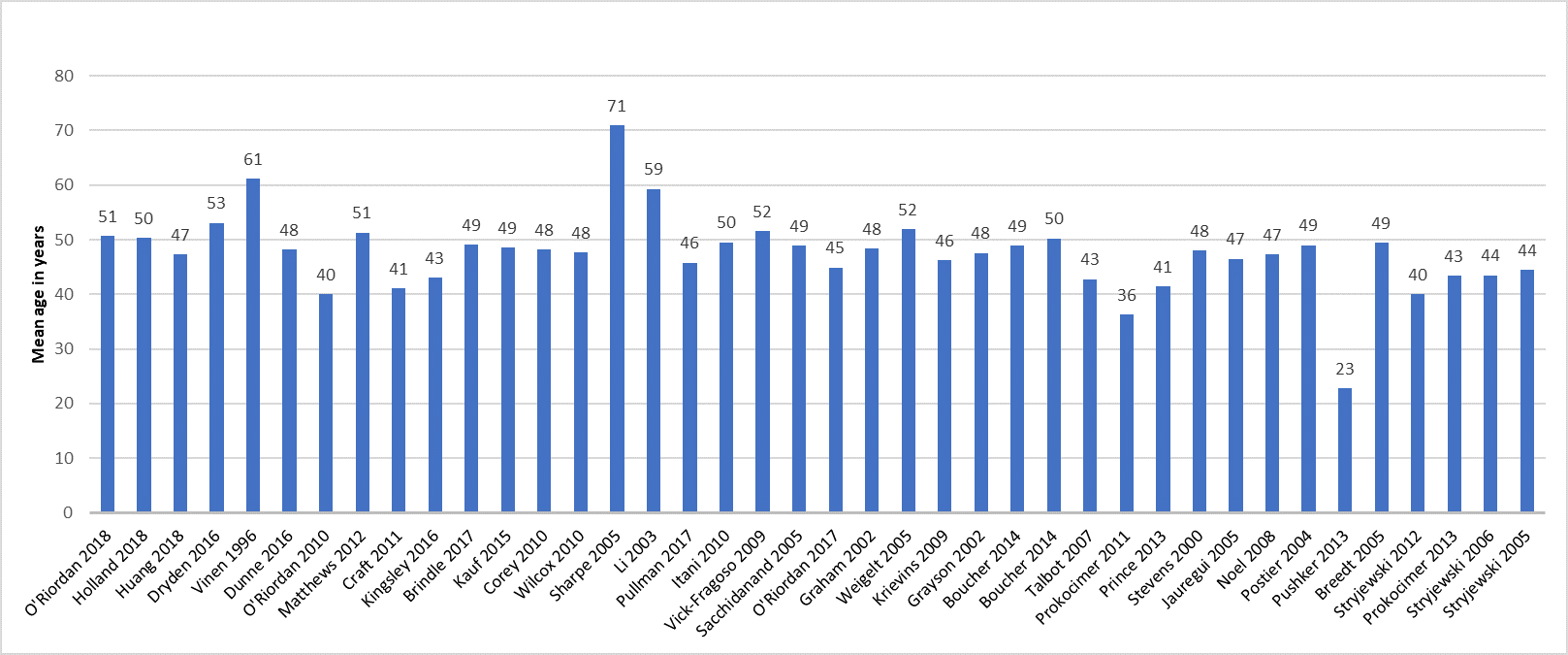


Appendix C2: Mean BMI in kg/m^2^


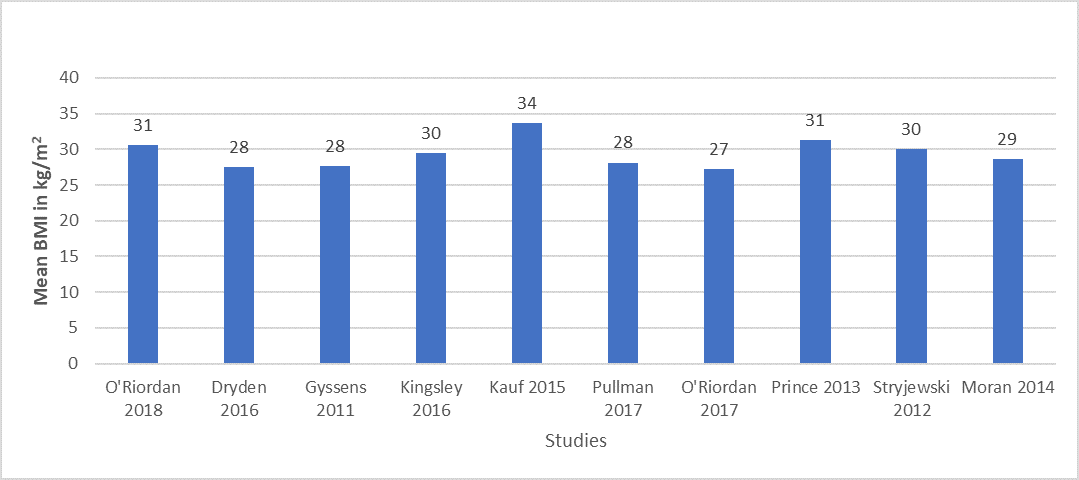


Appendix C3: Percentage of male patients


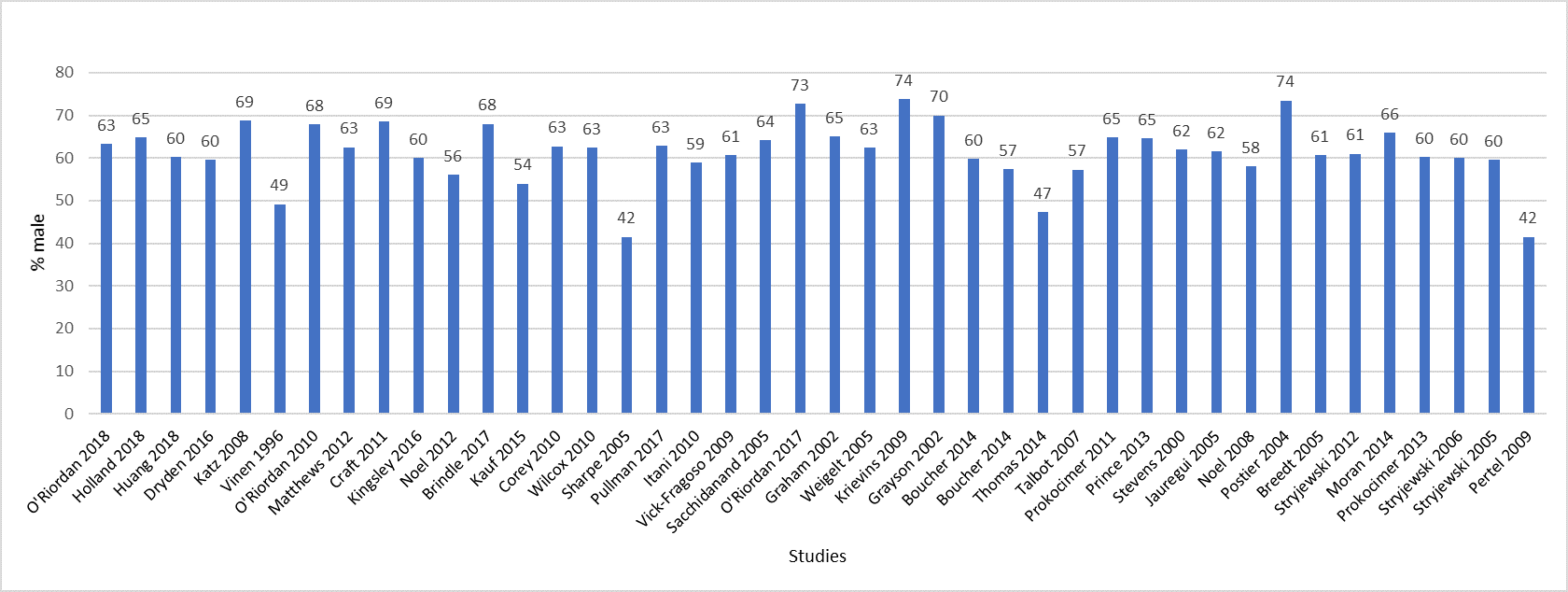


Appendix C4: Percentage of patients with different race


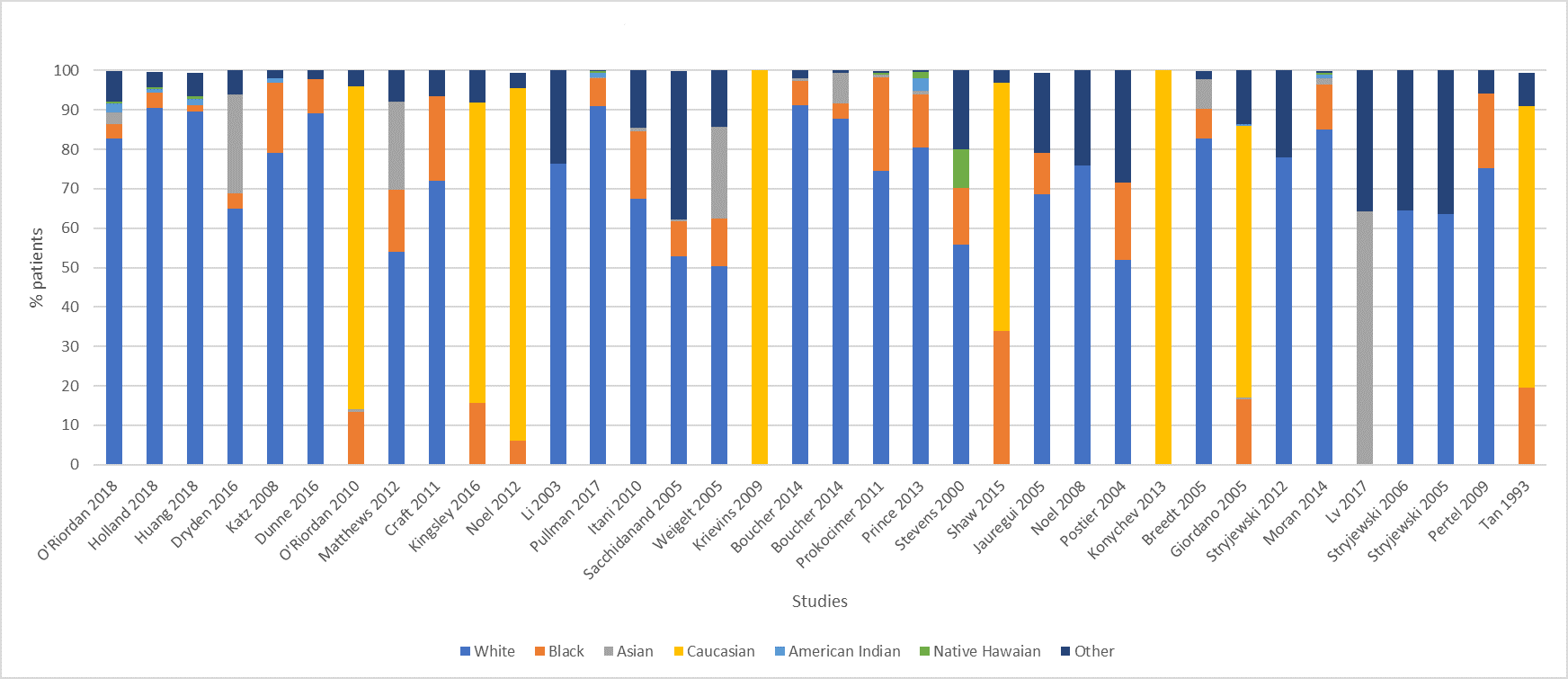


Appendix C5: Percentage of patients with Methicillin-susceptible *Staphylococcus aureus*


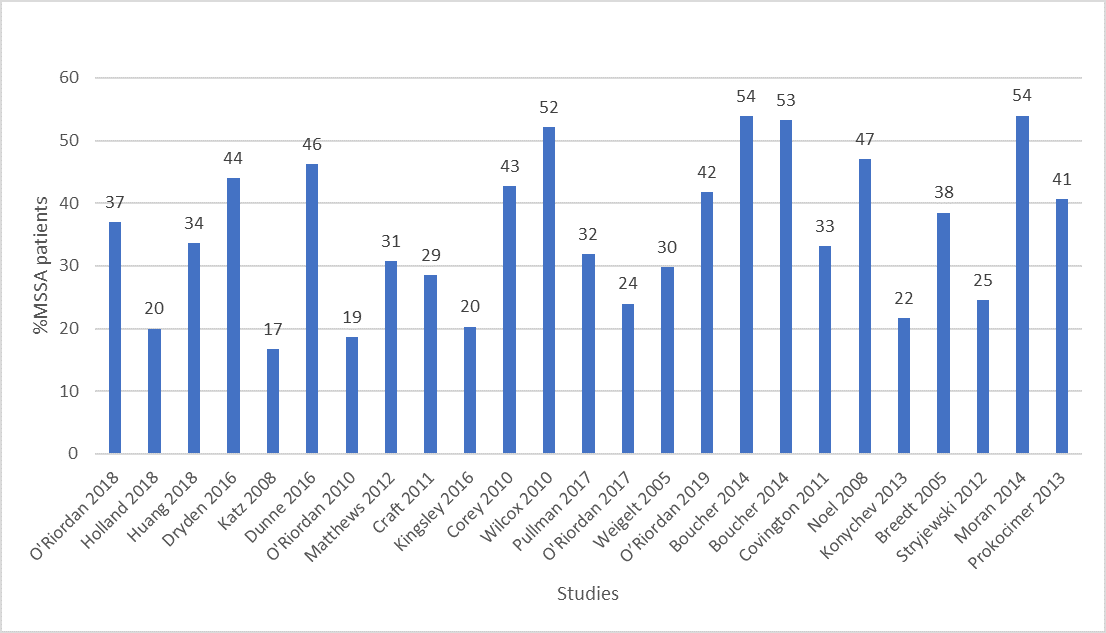
^MSSA: Methicillin-susceptible^ *^Staphylococcus aureus^*

Appendix C6: Percentage of patients with Methicillin-resistant *Staphylococcus aureus*


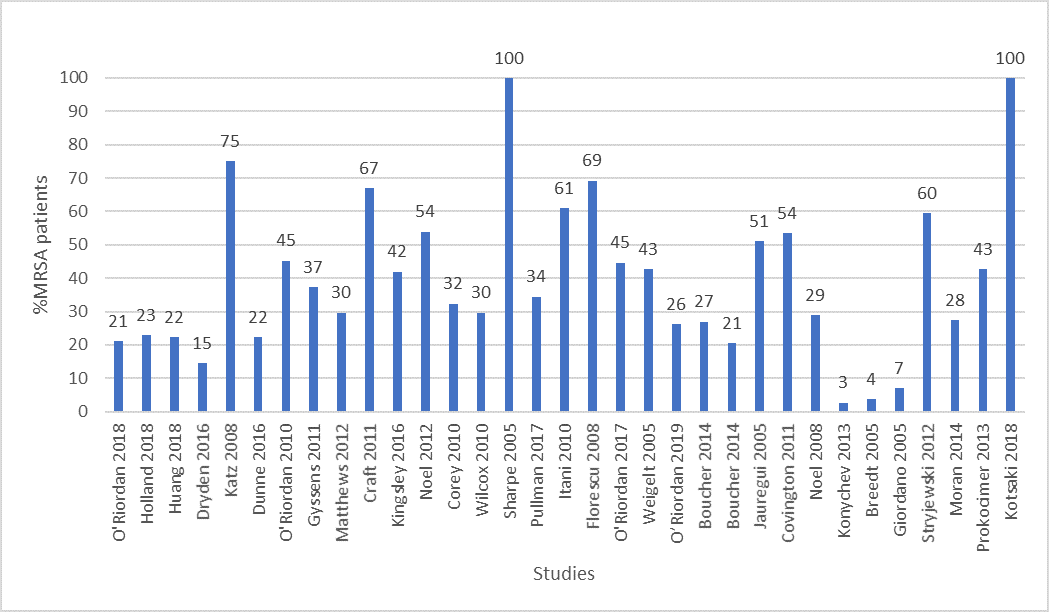


^MRSA: Methicillin-resistant^ *^Staphylococcus aureus^*

Appendix C7: Percentage of patients with Polymicrobial Infections


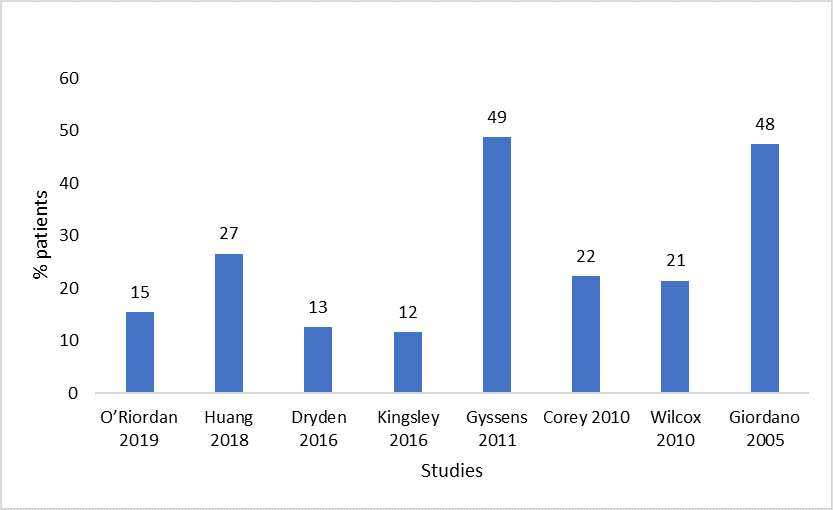


Appendix C8: Percentage of patients with Diabetes


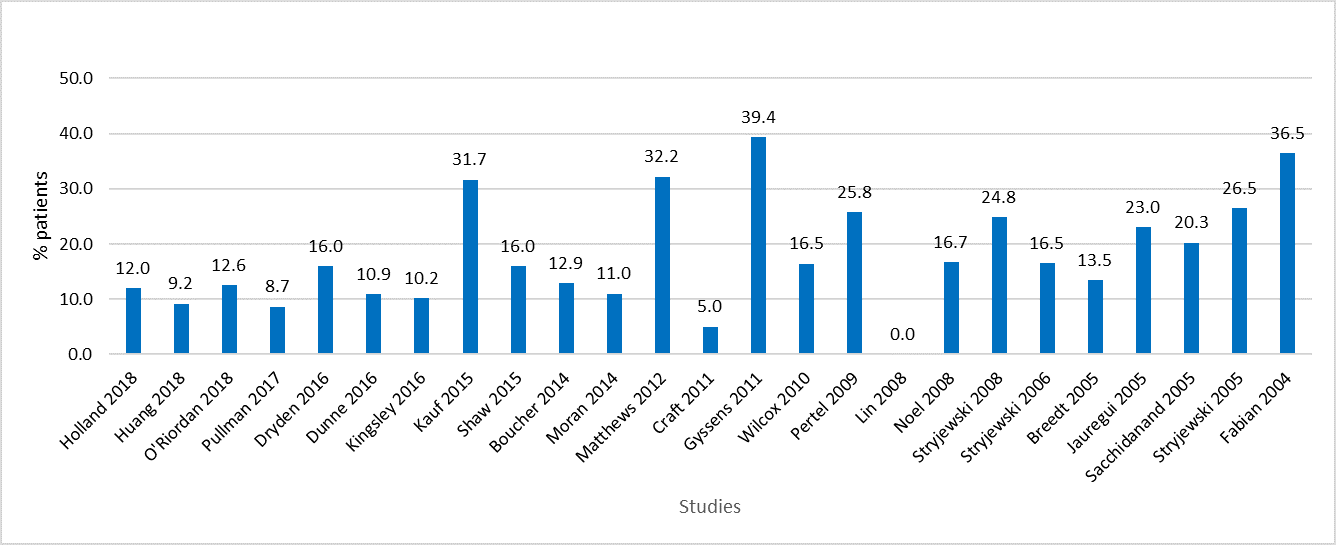


## **Appendix D: List of Included Studies**

| **Study** | **Infection subtype** | **Intervention (dose)** | **Comparator (dose)** | **Composite clinical response assessment timepoint** | **Early response assessment timepoint** | **Microbiological response assessment timepoint** | **Analysis population (s)** |
| --- | --- | --- | --- | --- | --- | --- | --- |
| ATLAS 1 [54] | cSSSI | TEL (10 mg/kg) | VAN (1 g) | 7-14 days after administration of the last dose of study medication | NA | NA | mITT |
| ATLAS 2 [54] | cSSSI | TEL (10 mg/kg) | VAN (1 g) | 7-14 days after administration of the last dose of study medication | NA | NA | mITT |
| CANVAS 1 [49] | cSSSI | CEF (600 mg) | VA (1 + 1 g) | 8-15 days after administration of the last dose of study medication | NA | NR | mITT, mITT |
| CANVAS 2 [50] | cSSSI | CEF (600 mg) | VA (1 g + 1 g) | 8-15 days after administration of the last dose of study medication | NA | NR | mITT, mITT |
| COVERS [55] | cSSTI | CEF (600 mg) | VA (15 mg/kg + 1 g) | 8-15 days after the last dose of study treatment | NA | NA | mITT |
| DAP-4 CELL-05-02 [56] | Cellulitis/ erysipelas | DAP (4 mg/kg) | VAN (according to ST) | 7-14 days after the last dose of study drug | NA | NA | mITT |
| DISCOVER 1 [57] | ABSSSI | DAL (1 g followed by 500 mg) | VL (1 g + 600 mg) | Day 14-15 (at the end of therapy) | 48-72 hours after the initiation of therapy | NA | PP, ITT |
| DISCOVER 2 [57] | ABSSSI | DAL (1 g followed by 500 mg) | VL (1 g + 600 mg) | Day 14-15 (at the end of therapy) | 48-72 hours after the initiation of therapy | NA | PP, ITT |
| ESTABLISH 1 [58] | ABSSSI | TED (200 mg) | LIN (600 mg) | 7-14 days after the end of treatment | 48-72 hours assessment after the first dose of study drug | NA | ITT, ITT |
| ESTABLISH-2 [59] | ABSSSI | TED (200 mg) | LIN (600 mg) | 7-14 days after the end of treatment | 48-72 hours visit | NA | ITT, ITT |
| FAST 1 [40] | cSSTI | TEL (7.5 mg/kg) | ST (VAN 1 g/NAF 2 g/OXA 2 g/CLOXA 0.5-1 g) | 7-14 days after the last dose of the study medication | NA | 7-14 days after the last dose of study medication | mITT, microE |
| FAST 2 [41] | cSSTI | TEL (10 mg/kg) | ST (VAN 1 g/NAF 2 g/OXA 2 g/CLOXA 0.5-1 g) | 7-14 days after the last dose of the study medication | NA | 7-14 days after administration of the last dose of the study medication | mITT, microE |
| OASIS 1 [60] | ABSSSI | OMA (100 mg) | LIN (600 mg) | 7-14 days after the last dose of a trial drug | 48-72 hours after first dose of study drug | NA | mITT, mITT |
| OASIS 2 [61] | ABSSSI | OMA (450 mg) | LIN (600 mg) | 7-14 days after last dose | 48-72 hours after first dose | NA | mITT, mITT |
| REVIVE 1 [62] | ABSSSI | ICL (80 mg) | VAN (15 mg/kg) | 7-14 days post-end of therapy | 48–72 hours after the start of administration of the study drug | NA | mITT, mITT |
| REVIVE 2 [63] | ABSSSI | ICL (80 mg) | VAN (15 mg/kg) | 7-14 days post-end of therapy | 48-72 hours | NA | mITT, mITT |
| Breedt 2005 [64] | cSSSI | TIG (50 mg) | VA (1 g + 2 g) | 12-92 days after last dose of study | NA | 12-92 days after last dose of study | mITT, microE |
| Craft 2011 [65] | ABSSSI | FUS (600 mg) | LIN (600 mg) | 7-14 days after end of therapy | Day 3 | NA | ITT, ITT |
| Itani 2010 [45] | cSSTI | LIN (600 mg) | VAN (15 mg/kg) | NA | NA | NA |  |
| Jauregui 2005 [66] | cSSSI | DAL (1 g followed by 500 mg) | LIN (600 mg) | 14 ± 2 days after completion of treatment with the study medication | Within 3 days after completion of treatment with the study medication | 14 ± 2 days after completion of treatment with the study medication | CE, CE, microE |
| Kauf 2015 [46] | cSSSI | DAP (4 mg/kg) | VAN (dosed as per investigator’s discretion) | NA | Day 3 | NA | Eva |
| Kingsley 2016 [53] | ABSSSI | DEL (300 mg); LIN (600 mg) | VAN (15 mg/kg) | Day 14+1 and ≥12 h after the final study drug dose | 48-72 hour after start of treatment | NR | ITT, ITT, microE |
| Krievins 2009 [67] | cSSSI | ICL (0.8 mg/kg); ICL (1.6 mg/kg) | VAN (1 g) | Day 30 | NA | Day 30 | mITT, microE |
| Lin 2008 [68] | cSSTI | LIN (60 mg) | VAN (1 g) | 7-28 days post-treatment | Within 72 hours after the last dose of study medication | 7-28 days post-treatment | Eva, Eva, microE |
| Matthews 2012 [42] | cSSSI | TIG (50 mg) | ASAC (15 g + 3 g or 1.2 g) | 8-50 days followed the end of therapy | NA | 8-50 days followed the end of therapy | mITT, microE |
| Noel 2008 [69] | cSSSI | CEFT (500 mg) | VAN (1 g) | 7-14 days after end of therapy | NA | 7-14 days after the end of therapy visit | ITT, ITT |
| Noel 2012a [43] | cSSSI | OMA (100 mg) | LIN (600 mg) | 10-17 days after the last dose of the treatment | NA | NA | mITT |
| Noel 2012b [70] | cSSTI | OMA (100 mg IV/300 mg Oral) | LIN (600 mg) | 10-17 days after completing therapy | NA | NA | ITT |
| O'Riordan 2015 [52] | cSSSI | DEL (300 mg); DEL (450 mg) | TIG (50 mg) | 14–21 days after the final dose of study drug | NA | NA | mITT |
| O'Riordan 2018 [27] | ABSSSI | DEL (300 mg) | VA (15 mg/kg + 2 g) | Day 14 ± 1 | 48-72 hours after initiation of treatment | Day 14 ± 1 | ITT, ITT, ITT |
| Pullman 2017 [28] | ABSSSI | DEL (300 mg) | VA (15 mg/kg + 2 g) | Day 14 ± 1 | 48-72 hours after treatment initiation | NA | ITT, ITT |
| Sacchidanand 2005 [71] | cSSSI | TIG (50 mg) | VA (1 g + 2 g) | 12 to 92 days following the end of treatment | NA | NA | mITT |
| Sharpe 2005 [36] | cSSTI | LIN (600 mg) | VAN (1 g) | NA | NA | NA |  |
| Stevens 2000 [72] | cSSTI | LIN (600 mg) | OD (2 g + 500 mg) | 15-21 days following treatment | NA | 15-21 days following treatment | mITT, microE |
| Talbot 2007 [51] | cSSSI | CEF (600 mg) | VAN (1 g) | 8-14 days after administration of the last dose of the study drug | NA | 8-14 days after administration of the last dose of the study drug | mITT, microE |
| Weigelt 2005 [44] | cSSTI | LIN (600 mg) | VAN (1 g) | 7 days after the end of therapy | NA | NA | mITT |
| Wilcox 2009 [37] | cSSSI | LIN (600 mg) | VAN (1 g) | 1-2 weeks after treatment | NA | 1-2 weeks after treatment | Modified mITT, modified microE |

^ABSSSI, acute bacterial skin and skin structure infection; ASAC, ampicillin/sulbactam or amoxicillin/clavulanate; CE, clinically evaluable; CEF, ceftaroline fosamil; CEFT, ceftobiprole; cSSSI, complicated skin and skin structure infection; cSSTI, complicated skin and skin tissue infection; DAL, dalbavancin; DAP, daptomycin; DEL, delafloxacin; Eva, evaluable; FUS, fusidic acid; ICL, iclaprim; ITT, intention-to-treat; LIN, linezolid; microE, microbiologically evaluable; microITT, microbiologically intention-to-treat; mITT, modified intention-to-treat; NA, not applicable; NAF, nafcillin; NR, not reported; OD, oxacillin + dicloxacillin; OMA, omadacycline; PP, per-protocol; ST, standard of care; TED, tedizolid; TEL, telavancin; TIG, tigecycline; VA, vancomycin + aztreonam; VAN, vancomycin; VL, vancomycin + linezolid^

## **Appendix E: Model fit for the fixed effect and random effect models**

|  | **FE model** | | | **RE model** | | |
| --- | --- | --- | --- | --- | --- | --- |
| **Analysis** | **Dbar** | **pD** | **DIC** | **Dbar** | **pD** | **DIC** |
| All patients CCR | 410.58 | 51.02 | 461.60 | 410.74 | 53.26 | 464.00 |
| All patients ER | 192.64 | 24.96 | 217.60 | 191.15 | 27.45 | 218.60 |
| All patients MR | 135.57 | 20.83 | 156.40 | 135.76 | 22.54 | 158.30 |
| MRSA CCR | 140.48 | 24.62 | 165.10 | 139.67 | 26.83 | 166.50 |
| MRSA MR | 112.98 | 18.82 | 131.80 | 110.12 | 22.08 | 132.20 |
| OBESE CCR | 25.94 | 5.01 | 30.95 | 25.96 | 5.03 | 30.99 |

^CCR, composite clinical response; DIC, deviance information criterion; ER, early response; FE, fixed effect; MR, microbiological response; MRSA, methicillin-resistant Staphylococcus aureus; RE, random effect^

## **Appendix F: Random effect models results**

All patients: composite clinical response


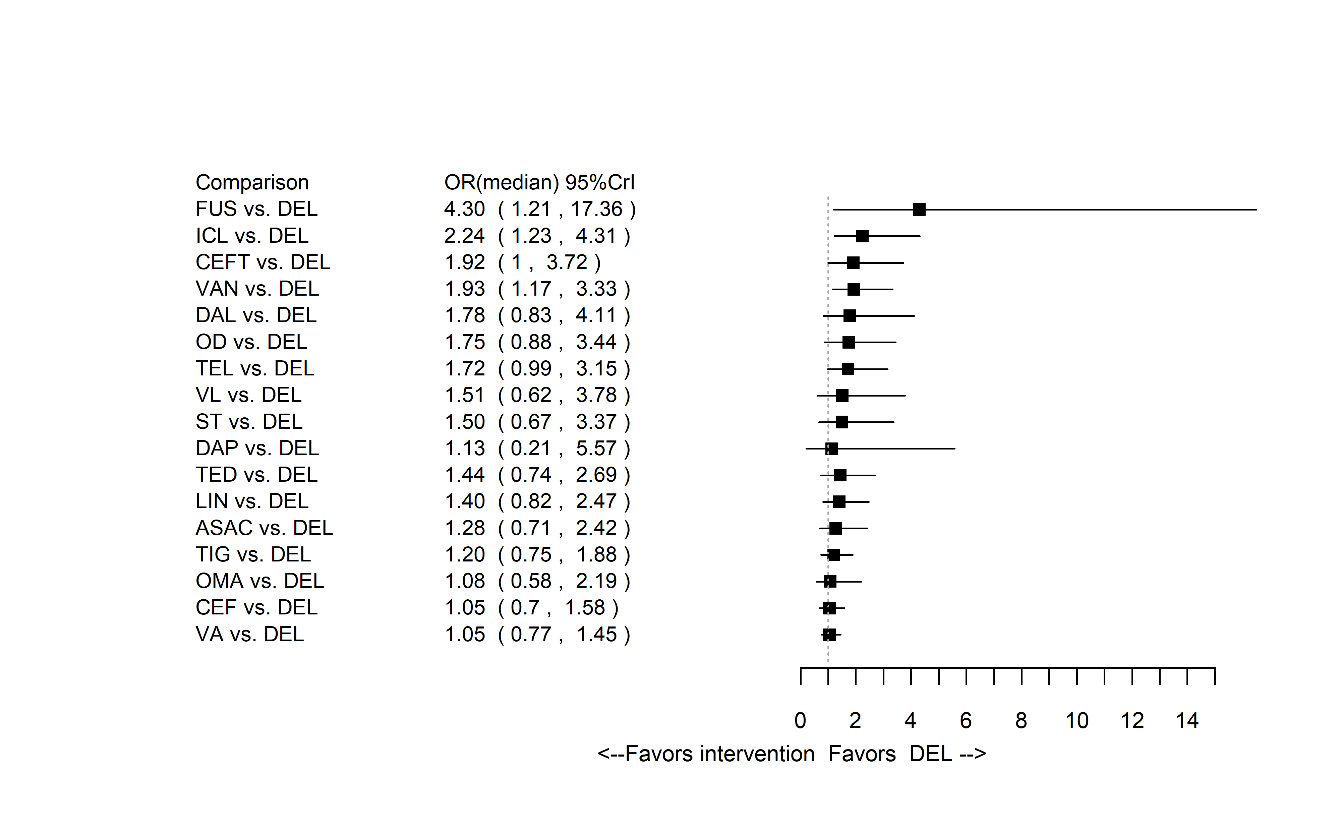


^ASAC, ampicillin + sulbactam or amoxycillin + clavulanate; CEF, ceftaroline fosamil, CEFT, ceftobiprole; DAL, dalbavancin; DAP, daptomycin, DEL: delafloxacin; FUS, fusidic acid; ICL, iclaprim; LIN, linezolid; VAN, vancomycin; OD, oxacillin + dicloxacillin; OMA, omadacycline; ST, standard of care, TED, tedizolid, TEL, telavancin; TIG, tigecycline, VA, vancomycin + aztreonam; VL, vancomycin + linezolid^

All patients: early response
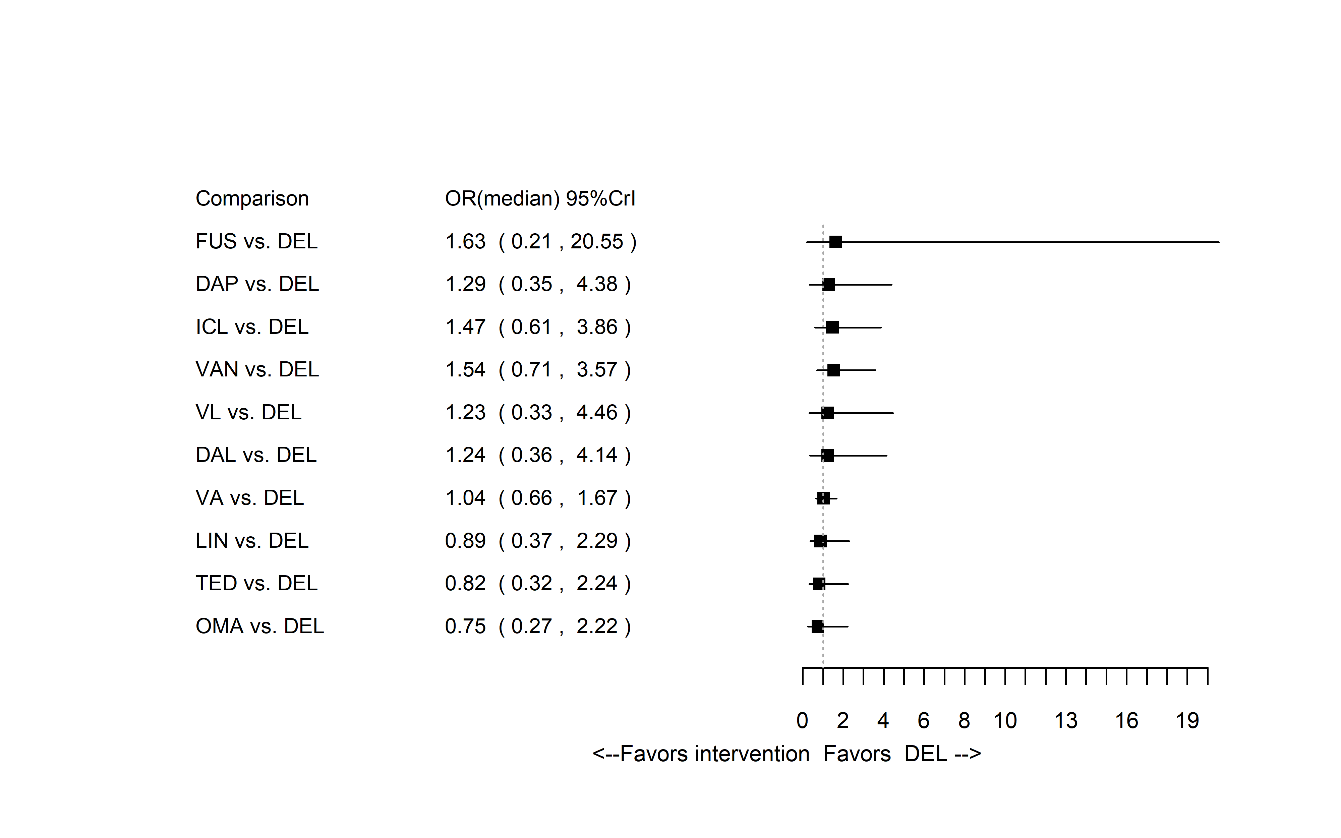


^DAL, dalbavancin; DAP, daptomycin; DEL, delafloxacin; FUS, fusidic acid; ICL, iclaprim; LIN, linezolid; OMA, omadacycline; TED, tedizolid; VA, vancomycin + aztreonam; VAN, vancomycin; VL, vancomycin + linezolid^

All patients: microbiological response
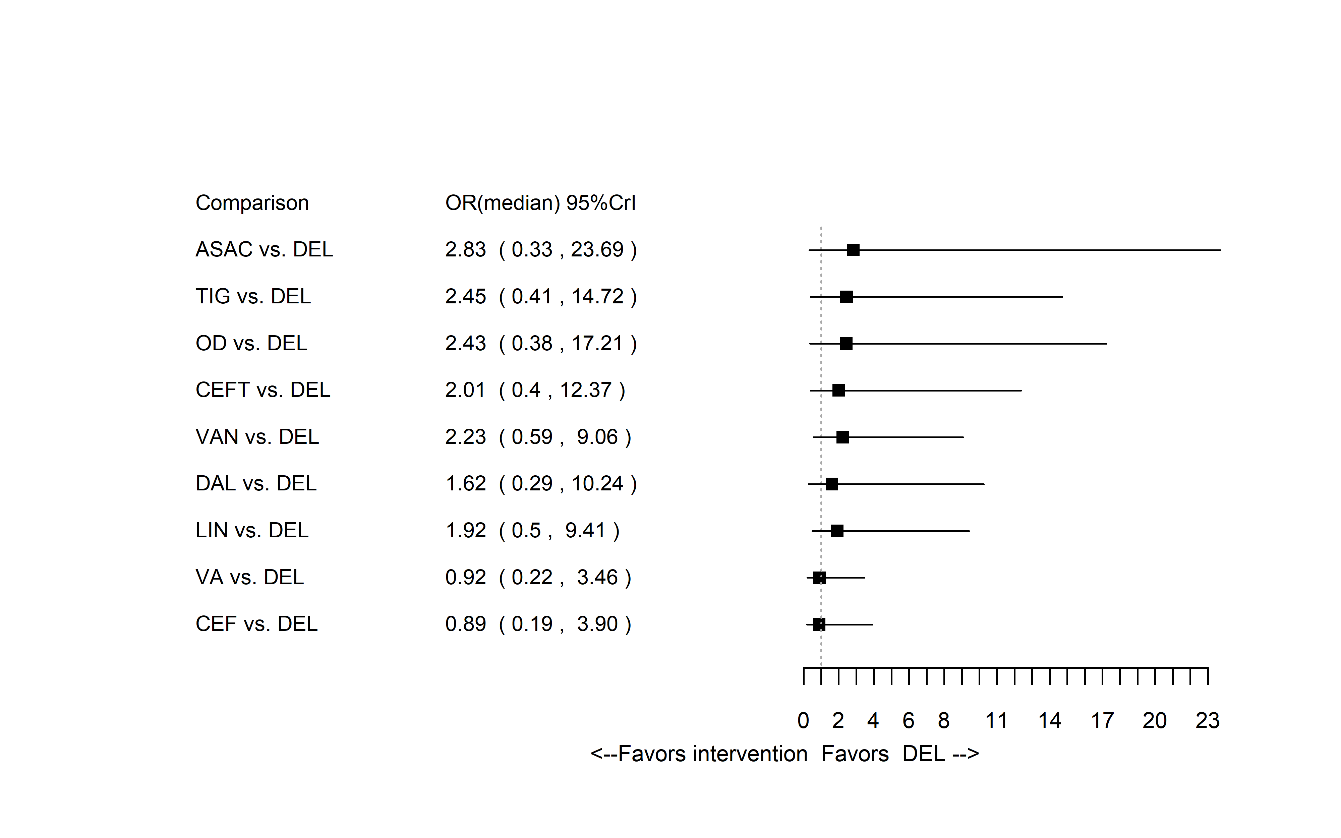


*^ASAC, ampicillin + sulbactam or amoxycillin + clavulanate; CEF, ceftaroline fosamil; CEFT, ceftobiprole; DAL, dalbavancin; DEL, delafloxacin; LIN, linezolid; OD, oxacillin + dicloxacillin; TIG, tigecycline; VA, vancomycin + aztreonam; VAN, vancomycin^*

Obese patients: compositive clinical response


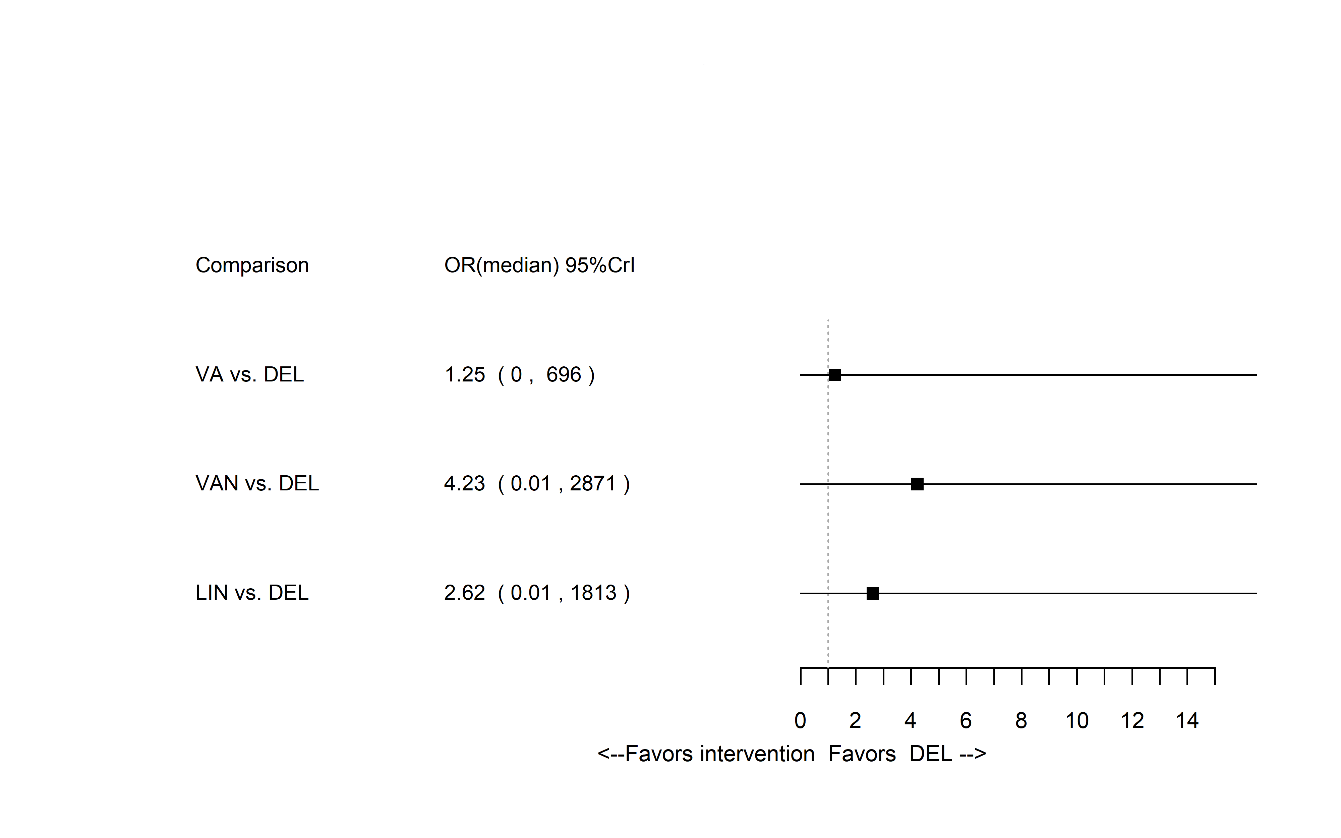


^DEL, delafloxacin; Lin, linezolid; VA, vancomycin + aztreonam; VAN, vancomycin^

## **Appendix G: Inconsistency assessment findings**

All patients: composite clinical response

| **Treatment difference (log-odds)** | **Consistency model** | | | **Inconsistency model** | | |
| --- | --- | --- | --- | --- | --- | --- |
|  | **Median** | **2.5%** | **97.5%** | **Median** | **2.5%** | **97.5%** |
| VAN, VA | 0.58 | 0.01 | 1.14 | 0.41 | -67.41 | 195.00 |
| VAN, CEF | 0.57 | -0.02 | 1.16 | 0.53 | 0.12 | 1.70 |
| VAN, TIG | 0.44 | -0.20 | 1.07 | 0.02 | -67.13 | 193.70 |
| VAN, DEL | 0.64 | 0.11 | 1.15 | 0.66 | 0.46 | 1.25 |
| VA, CEF | -0.01 | -0.24 | 0.22 | -0.01 | -0.09 | 0.23 |
| VA, TIG | -0.14 | -0.44 | 0.16 | -0.13 | -0.24 | 0.18 |
| VA, DEL | 0.06 | -0.21 | 0.33 | 0.05 | -0.05 | 0.33 |
| CEF, TIG | -0.13 | -0.51 | 0.25 | 0.65 | -66.61 | 194.30 |
| CEF, DEL | 0.07 | -0.28 | 0.41 | 0.53 | -67.41 | 198.20 |
| TIG, DEL | 0.19 | -0.20 | 0.60 | 0.26 | -0.28 | 1.79 |

^CEF, ceftaroline fosamil; DEL: delafloxacin; VAN, vancomycin; TIG, tigecycline, VA, vancomycin + aztreonam^

All patients: microbiological response

| **Treatment difference (log-odds)** | **Consistency model** | | | **Inconsistency model** | | |
| --- | --- | --- | --- | --- | --- | --- |
|  | **Median** | **2.5%** | **97.5%** | **Median** | **2.5%** | **97.5%** |
| VAN, VA | 0.81 | -0.52 | 2.21 | -0.45 | -195.90 | 195.60 |
| VAN, CEF | 0.86 | -0.47 | 2.26 | 1.27 | -0.68 | 3.52 |
| VAN, DEL | 0.76 | -0.29 | 1.96 | 0.64 | -0.50 | 2.00 |
| VA, CEF | 0.05 | -0.26 | 0.35 | 0.04 | -0.27 | 0.35 |
| VA, DEL | -0.03 | -1.18 | 1.08 | 0.09 | -1.23 | 1.44 |
| CEF, DEL | -0.08 | -1.26 | 1.07 | 0.51 | -196.90 | 197.30 |

^CEF, ceftaroline fosamil, DEL: delafloxacin; VAN, vancomycin; VA, vancomycin + aztreonam^

## **Appendix H: Networks of evidence for all patients: early clinical response**

**
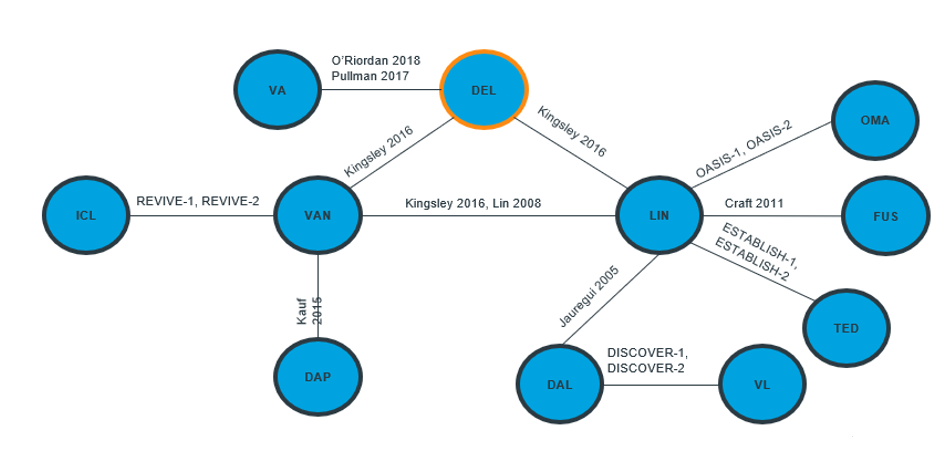
**

^DAL, dalbavancin; DAP, daptomycin; DEL, delafloxacin; FUS, fusidic acid; ICL, iclaprim; LIN, linezolid; OMA, omadacycline; TED, tedizolid; VA, vancomycin + aztreonam; VAN, vancomycin; VL, vancomycin + linezolid^

## **Appendix I: Network of evidence for all patients: microbiological response**


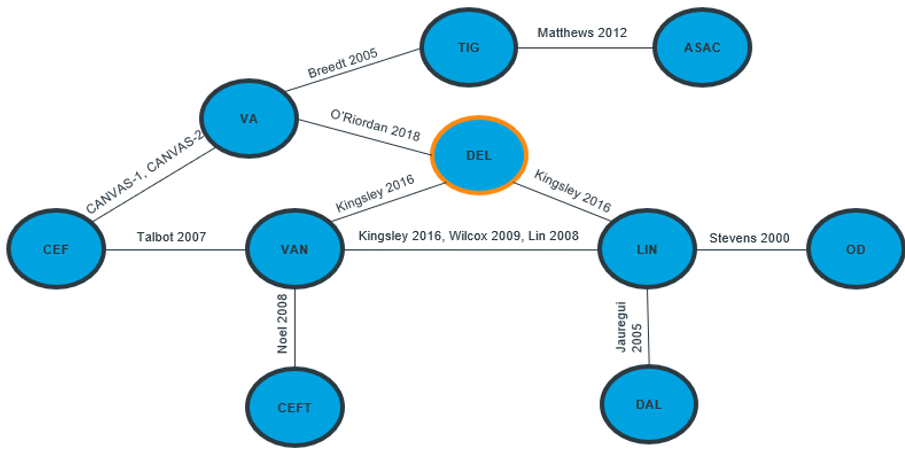


^ASAC, ampicillin/sulbactam or amoxicillin/clavulanate; CEF, ceftaroline; CEFT, ceftobiprole; DAL, dalbavancin; DEL, delafloxacin; LIN, linezolid; OD, oxacillin + dicloxacillin; TIG, tigecycline; VA, vancomycin + aztreonam; VAN, vancomycin^

## **Appendix J: Network of evidence for obese subpopulation: composite clinical response**


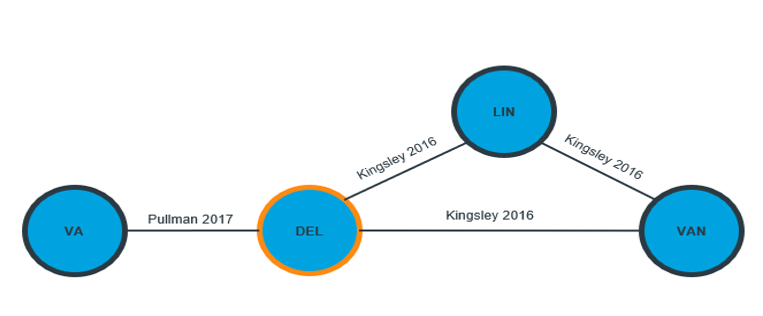


^DEL, delafloxacin; LIN, linezolid; VA, vancomycin + aztreonam; VAN, vancomycin^

## **Appendix K: Forest plot for patients with MRSA infection: composite clinical response**

**
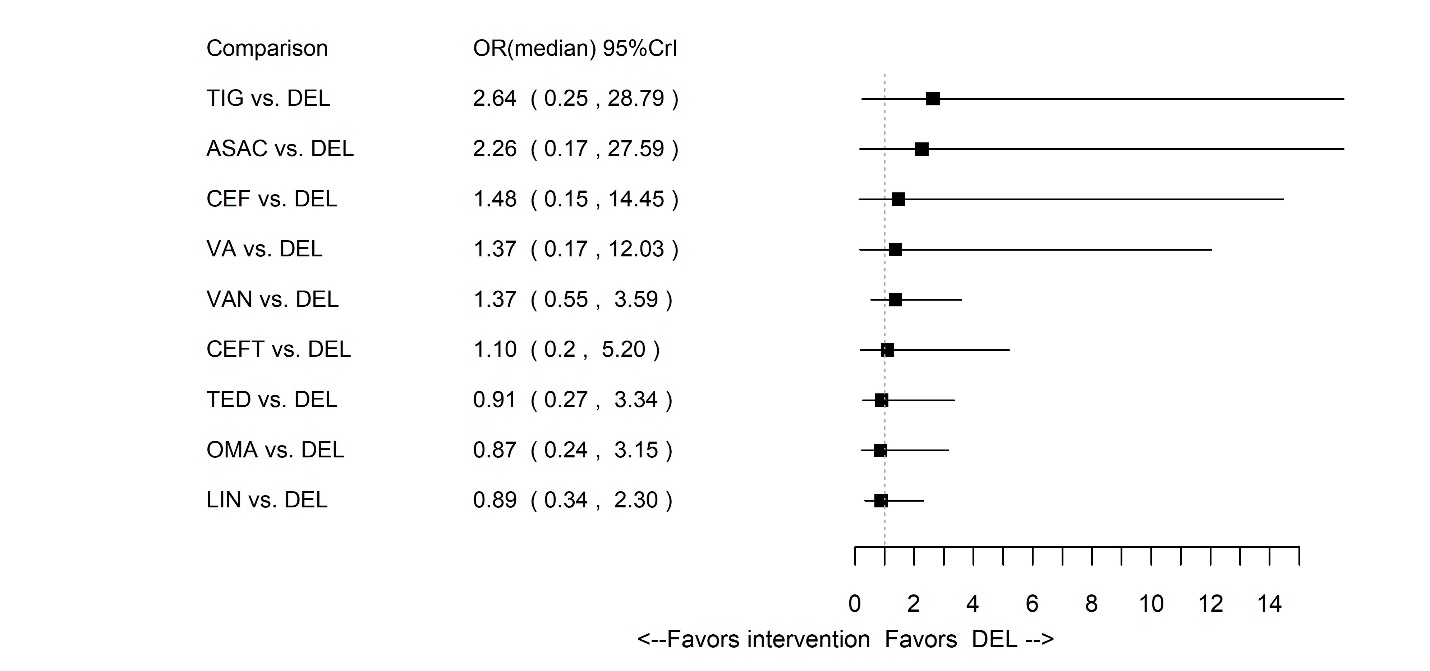
**

^ASAC, ampicillin/sulbactam or amoxicillin/clavulanate; CEF, ceftaroline; CEFT, ceftobiprole; DEL, delafloxacin; LIN, linezolid; MRSA: Methicillin-resistant^ *^Staphylococcus aureus;^*^OMA, omadacycline; TED, tedizolid; TIG, tigecycline; VA, vancomycin + aztreonam; VAN, vancomycin^

## **Appendix L: Forest plot for patients with MRSA infection: microbiological response**


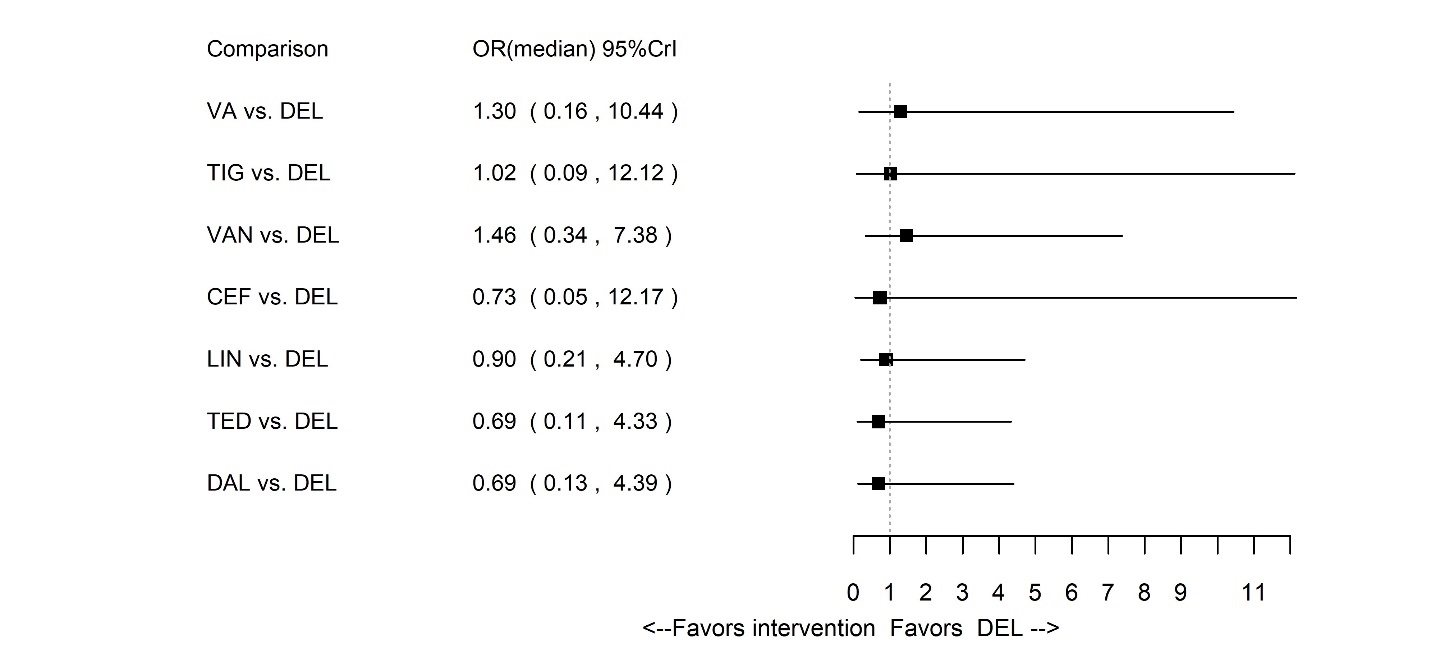


^CEF, ceftaroline; DAL, dalbavancin, DEL, delafloxacin; LIN, linezolid; MRSA: Methicillin-resistant^ *^Staphylococcus aures;^* ^TED, tedizolid; TIG, tigecycline; VA, vancomycin + aztreonam; VAN, vancomycin^
